# Interpretable Machine Learning Model for Predicting and Assessing the Risk of Diabetic Nephropathy: Prediction Model Study

**DOI:** 10.2196/64979

**Published:** 2025-10-22

**Authors:** Yili Wen, Zhiqiang Wan, Huiling Ren, Xu Wang, Weijie Wang

**Affiliations:** 1 Institute of Medical Information/Medical Library, Chinese Academy of Medical Sciences & Peking Union Medical College, 3 Yabao Road, Chaoyang District, Beijing, 100010, China, 86 01052328911; 2 Peking Union Medical College Hospital, Chinese Academy of Medical Science & Peking Union Medical College, Beijing, China

**Keywords:** type 2 diabetes, machine learning, interpretability analysis, ML, diabetes, risk assessment, diabetic nephropathy, hypertension, renal disease, renal function, glucose, oxidative stress, inflammation, fibrosis, kidney, quality of life, ML model, predictive tool, early diagnosis, patient outcomes

## Abstract

**Background:**

Diabetic nephropathy (DN), a severe complication of diabetes, is characterized by proteinuria, hypertension, and progressive renal function decline, potentially leading to end-stage renal disease. The International Diabetes Federation projects that by 2045, 783 million people will have diabetes, with 30%‐40% of them developing DN. Current diagnostic approaches lack sufficient sensitivity and specificity for early detection and diagnosis, underscoring the need for an accurate, interpretable predictive model to enable timely intervention, reduce cardiovascular risks, and optimize health care costs.

**Objective:**

This study aimed to develop and validate a machine learning–based predictive model for DN in patients with type 2 diabetes, with a focus on achieving high predictive accuracy while ensuring transparency and interpretability through explainable artificial intelligence techniques, thereby supporting early diagnosis, risk assessment, and personalized clinical decision-making.

**Methods:**

Our retrospective cohort study investigated 1000 patients with type 2 diabetes using data from electronic medical records collected between 2015 and 2020. The study design incorporated a sample of 444 patients with DN and 556 without, focusing on demographics, clinical metrics such as blood pressure and glucose levels, and renal function markers. Data collection relied on electronic records, with missing values handled via multiple imputation and dataset balance achieved using Synthetic Minority Oversampling Technique (SMOTE). In this study, advanced machine learning algorithms, namely Extreme Gradient Boosting (XGBoost), CatBoost, and Light Gradient-Boosting Machine (LightGBM), were used due to their robustness in handling complex datasets. Key metrics, including accuracy, precision, recall, *F*_1_-score, specificity, and area under the curve, were used to provide a comprehensive assessment of model performance. In addition, explainable machine learning techniques, such as Local Interpretable Model-Agnostic Explanations (LIME) and Shapley Additive Explanations (SHAP), were applied to enhance the transparency and interpretability of the models, offering valuable insights into their decision-making processes.

**Results:**

XGBoost and LightGBM demonstrated superior performance, with XGBoost achieving the highest accuracy of 86.87%, a precision of 88.90%, a recall of 84.40%, an *F*_1_-score of 86.44%, and a specificity of 89.12%. LIME and SHAP analyses provided insights into the contribution of individual features to elucidate the decision-making processes of these models, identifying serum creatinine, albumin, and lipoproteins as significant predictors.

**Conclusions:**

The developed machine learning model not only provides a robust predictive tool for early diagnosis and risk assessment of DN but also ensures transparency and interpretability, crucial for clinical integration. By enabling early intervention and personalized treatment strategies, this model has the potential to improve patient outcomes and optimize health care resource usage.

## Introduction

### Background

Diabetic nephropathy (DN), severe microvascular complications of diabetes, primarily manifests as proteinuria, hypertension, and a progressive decline in renal function, potentially leading to end-stage renal disease. The pathogenesis of DN is attributed to a high-glucose milieu, oxidative stress, inflammation, and fibrosis, collectively contributing to substantial morphological changes in kidneys including thickening of glomerular basement membrane, glomerulosclerosis, tubular atrophy, interstitial inflammation, and renal fibrosis [1]. The prevalence of diabetes and diabetic kidney disease has been increasing, and the International Diabetes Federation projected that the number of patients with diabetes would rise to 783 million by 2045. Notably, approximately 30%‐40% of these patients are expected to develop DN [2], with a mortality rate 30 times higher than that of diabetic patients without kidney disease [3]. Hence, the importance of early detection cannot be overstated in managing DN. Early diagnosis not only significantly reduces the reliance on costly medical resources such as dialysis and transplantation but also alleviates the economic burden on patients [4]. By intervening in the early stages of DN, clinicians can effectively preserve renal function and slow disease progression, thereby enhancing the quality of life and reducing the risk of cardiovascular complications, a major cause of death among patients with diabetes. Moreover, from a perspective of health economics, early detection is crucial as it reduces the need for intensive later-stage treatments, allowing for the reallocation of medical resources to other pressing needs [5]. Consequently, investing in the early detection of DN not only benefits patients but also enhances the efficiency of health care resource usage across society.

Machine learning (ML), as a significant branch of artificial intelligence (AI), has revolutionized the field of medical research by analyzing complex datasets to discover models and make predictions. Particularly in disease prediction and classification, ML algorithms can handle vast amounts of clinical and biological data, identify risk factors, predict disease onset, and accurately classify disease subtypes [6]. In contrast, traditional statistical methods often perform poorly when dealing with the high dimensionality and nonlinearity of biomedical data, whereas ML algorithms, such as decision trees, random forests, support vector machines, and neural networks, exhibit advantages that traditional statistical methods cannot match [7]. ML algorithms have been widely applied in clinical research and shown outstanding performance in various fields. For example, they have achieved significant results in the early prediction of acute kidney injury [8], malaria prediction [9], and cervical cancer survival prediction [10]. Despite the tremendous potential of ML models in predictive analysis, their application in clinical environments is often hindered by the “black box” nature of many algorithms. This opacity limits clinicians’ understanding, trust, and effective use of ML predictions. To address this issue, explainable machine learning (XML) techniques have emerged, aiming to enhance the transparency and interpretability of models [11]. By using techniques such as Shapley Additive Explanations (SHAP) and Local Interpretable Model-Agnostic Explanations (LIME), XML can elucidate the contribution of individual features to prediction outcomes, thereby increasing the model’s transparency and interpretability [12]. This interpretability is crucial for integrating ML into clinical workflows, as it allows health care providers to validate model outputs based on clinical knowledge, explain decisions to patients, and comply with regulatory standards [13]. Ultimately, XML holds the promise of bridging the gap between advanced analytics and clinical applications, fostering more informed and more confident decision-making in patient care.

### Objective

**Figure 1.**
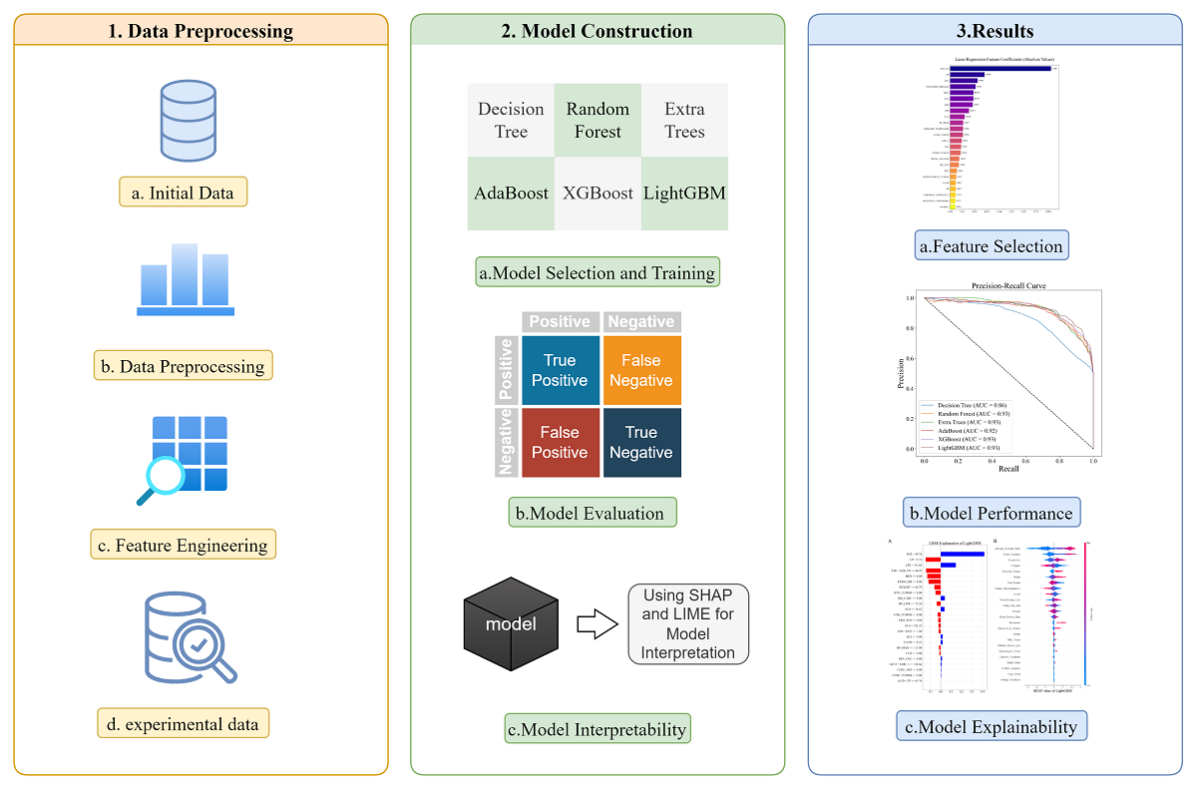
Overview of the research framework. This diagram illustrates the complete workflow from data preprocessing to model interpretability, where the orange module represents the data processing stage, the green module denotes the model construction phase, and the blue module signifies the result analysis stage. The directional arrows indicate the sequential order of processes. AdaBoost: Adaptive Boosting; AUC: area under the curve; LightGBM: Light Gradient-Boosting Machine; LIME: Local Interpretable Model-Agnostic Explanations; SHAP: Shapley Additive Explanations; XGBoost: Extreme Gradient Boosting.

In the clinical management of DN, early diagnosis and precise treatment are crucial for improving patient outcomes. However, traditional diagnostic methods often fall short in predicting the complex progression of the disease, necessitating new tools to enhance predictive accuracy and reliability. This study aims to develop and validate an ML-based model for predicting DN, emphasizing both high predictive accuracy and model interpretability to meet clinicians’ needs for transparency. By addressing the gap in both predictive performance and interpretability, this model provides a more holistic approach to managing DN. By creating a new predictive tool, we aim to provide clinicians with a deep understanding of the model’s predictive logic, thereby enhancing trust and application of the predictions. We meticulously designed and integrated various ML algorithms, including decision trees, random forests, extra trees, Adaptive Boosting (AdaBoost), Extreme Gradient Boosting (XGBoost), and Light Gradient-Boosting Machine (LightGBM), to build a model with significant predictive accuracy. Concurrently, we used LIME and SHAP methods for in-depth analysis of the model’s interpretability, ensuring transparency and fairness in the prediction process. The core contribution of this study lies in enhancing model interpretability, thereby increasing its credibility and practicality in real medical applications. This model not only provides a scientifically transparent decision support system for early diagnosis, risk assessment, and personalized treatment of DN but also aids doctors in devising more precise intervention strategies to improve patient outcomes. We believe that the generalizability and effectiveness of these methods will lay a solid foundation for the broader application of ML technologies in various medical scenarios and open new avenues for medical research. We outline the overall approach of our study as shown in Figure 1.

### Related Work

XML has been introduced to enhance model transparency and reliability, helping clinicians better understand the predictions made by these models. Several studies have successfully demonstrated how interpretability techniques can improve the transparency of clinical decision-making. Chadaga et al [14] used SHAP and LIME to predict COVID-19 prognosis using clinical markers, aiming to identify high-risk patients early and provide appropriate treatments to prevent severe outcomes, while also making AI predictions interpretable and trustworthy for medical professionals. Khanna et al [15] built a decision support system using SHAP and LIME to predict osteoporosis risk, aiming to enhance early diagnosis and treatment while making ML models interpretable and reliable for medical professionals. Guan et al [16] used SHAP to interpret venous thromboembolism risks in patients who are critically ill, with the goal of improving early risk identification, enabling timely interventions, and fostering transparency and trust in the decision-making process. Zhong et al [17] introduced SHAP to significantly improve the accuracy of blood oxygen saturation estimation based on neck photoplethysmography, thereby enhancing the reliability of noninvasive oxygen monitoring. Suh et al [18] used LIME to analyze a deep learning model for osteoporosis risk screening, identifying and ranking critical features that contribute to risk prediction. This approach enhances the interpretability of ML models, facilitating personalized health care and aiding clinicians in understanding the decision-making process. In the domain of XML, SHAP and LIME are among the most commonly used methods; however, other approaches, such as partial dependence plot (PDP), are also available. Bernard et al [19] used PDP to visualize the influence of individual features on the model’s output by isolating their effects while averaging over all other features. Zhang et al [20] used PDP to analyze the marginal effect of individual features on the model’s predictions by averaging their impacts while keeping other features constant. This method helps reveal how changes in a particular feature influence the predicted outcomes, providing clinicians with interpretable insights into feature significance and supporting informed decision-making in personalized health care.

## Methods

### Data Source

The dataset for this study originated from the National Population Health Data Center’s “Diabetes Complications Dataset” and comprised detailed records of 1000 patients with type 2 diabetes. The complete dataset of 1000 patients was used for our analysis without applying any additional inclusion or exclusion criteria, as the dataset had already undergone an initial selection and screening process before public release by the data provider. The dataset covers 87 features, including patients’ basic demographic information such as age, gender, ethnicity, and marital status, biochemical test results such as blood glucose levels, lipid analysis, renal function indicators, and other relevant hematological parameters, and comorbidities such as kidney disease, cardiovascular diseases, fatty liver, and many other chronic conditions. The dataset contained information on 444 patients with DN and 556 patients without nephropathy, providing a rich empirical basis for the study of DN risk prediction.

### Data Preprocessing

In this study, features with a data missing proportion exceeding 75% (750/1000) were excluded due to their severely limited informational content and minimal impact on the research outcomes. For features with a data missing proportion less than 75% (750/1000), multiple imputation was used. This method, based on multivariate regression models and iterative algorithms, is well-suited for handling clinical data with diverse feature types due to its ability to preserve interfeature correlations and prevent the disruption of internal data relationships, which is typical for singular imputation methods. The fundamental approach involves initially filling each feature containing missing values with simple methods such as the mean to provide initial values, followed by the use of current filled values from other features to predict and update missing values through regression models. This process iterates until the imputation converges or the predefined maximum number of iterations is reached. In addition, we noted that the ratio of patients with DN to those without is 444:556, close to 4:5. Although this imbalance is not particularly severe, we used the Synthetic Minority Oversampling Technique (SMOTE) to enhance the accuracy and reliability of our model predictions. SMOTE generates new synthetic samples by randomly interpolating between minority class samples and their nearest neighbors. This method not only increases the number of minority class samples but also maintains intraclass diversity, thereby avoiding the potential overfitting issues associated with simple sample replication. Through this approach, SMOTE has effectively addressed the issue of data imbalance, laying a solid foundation for the development of a more robust and more accurate predictive model.

### Statistical Analysis

This study used Python version 3.9.5, originally developed by Guido van Rossum and currently maintained by the Python Software Foundation, for data analysis. In the statistical data analysis, the Shapiro-Wilk test was first conducted on continuous variables to determine whether they follow a normal distribution. If these variables did not follow a normal distribution, the Mann-Whitney *U* test was used to explore their associations with DN. The Mann-Whitney *U* test helped examine whether there are significant statistical differences between the patient and nonpatient groups, making it an effective nonparametric testing method. If these variables followed a normal distribution, the Student *t* test was used to analyze their associations with DN. This parametric testing method assessed the significance of differences in the means of 2 samples, aiding in the identification of key indicators related to the risk of DN. For categorical variables, chi-square tests were used to analyze their associations with DN, detecting distribution differences of categorical variables across different disease states. Through these tests, we gained a more comprehensive understanding of how different types of data characteristics influence the risk of DN. Statistical significance was defined as *P*<.001.

### Feature Selection

Given the limited sample size and large number of features in the data involved in this study, we adopted the least absolute shrinkage and selection operator (lasso) method for feature selection to ensure the efficiency of the analysis and the accuracy of the results. Lasso introduces the L1 paradigm as a penalty term in regression analysis, making it widely used in high-dimensional data processing [21]. Its objective function is:


$$min_{\beta }\left\{\frac{1}{2n}\sum_{i=0}^{n}(y_{i}-\sum_{j=1}^{p}x_{ij}\beta_{j}{)}^{2}+\lambda \sum_{j=0}^{p}\beta_{j}\right\}$$

where, $y_{i}$ is the response variable of the ith observation, $x_{ij}$ is the jth feature of the ith observation, $\beta_{j}$ is the regression coefficient of the jth feature, λ is the regularization parameter used to control the complexity of the model, n is the number of samples, and p is the number of features.

By adjusting the regularization parameter λ, lasso achieves a balance between model complexity and fitting accuracy. The core mechanism is to drive some of the regression coefficients to zero through the L1 paradigm penalty, enabling variable selection and feature downscaling. This capability gives lasso a significant advantage when dealing with clinical data, as it is able to automatically screen out important variables and eliminate redundant or irrelevant features, making it particularly suitable for high-dimensional data with a large number of features. Through this regularization, lasso enhances the stability and predictive power of the model, preventing overfitting and ensuring more robust model performance on test sets. In addition, by retaining only the most predictive features, lasso significantly improves the accuracy of the model’s predictions.

### Model Construction

In this study, we used a series of powerful ensemble learning algorithms, including random forests, extra trees, AdaBoost, XGBoost, and LightGBM. These algorithms are widely used in a variety of ML applications due to their excellent predictive performance and efficient processing speed.

Decision tree is a fundamental ML algorithm used for classification and regression [22]. The data are split into different subsets based on a series of conditions, ultimately forming a tree structure. Each node represents a feature, each branch represents one possible value of the feature, and each leaf node represents a category or regression value.

Random forest is an ensemble learning method that enhances prediction accuracy and stability by constructing multiple decision trees [23]. The core idea is to train multiple decision trees using different subsets of data and features, and obtain the final result by voting or averaging the predictions of all trees. This approach effectively reduces overfitting and improves the model’s generalization ability.

Extra trees, or extremely randomized trees, is an improved random forest method that constructs decision trees by randomly selecting features and split points [24]. Unlike random forests, extra trees randomly selects multiple split points at each node, from which the best split point is chosen. This completely random strategy reduces the variance of the model and improves generalization while retaining the interpretability and training speed of the decision tree model.

AdaBoost is a boosting method based on an additive model that improves overall classification performance by progressively weighting the training of multiple weak classifiers [25]. In each round of training, misclassified samples are given higher weights, prompting subsequent classifiers to focus more on these difficult-to-classify samples. The final model is a weighted sum of multiple weak classifiers, which gradually reduces error and significantly enhances performance.

XGBoost is an efficient gradient-boosting decision tree algorithm that combines parallel processing and regularization techniques to improve the speed and performance of the model [26]. Key features include the use of second-order derivative information, the ability to handle missing values, and pruning operations on the decision tree. These features enable XGBoost to perform well when dealing with large-scale and high-dimensional data.

LightGBM is an efficient gradient-boosting framework that improves training speed and memory usage through a histogram-based decision tree learning algorithm [27]. Its features include support for categorical features, a depth-first strategy using leaf-count limitation, and efficient parallel processing. These characteristics allow LightGBM to have a significant advantage in handling large-scale data.

### Model Evaluation

To comprehensively evaluate the model’s performance, this study used multiple evaluation metrics, including accuracy, precision, recall, *F*_1_-score, specificity, and area under the curve (AUC). These metrics reflect various aspects of the model’s performance, ensuring an objective and thorough assessment.

Accuracy refers to the ratio of correctly predicted samples to the total number of samples and reflects the overall prediction accuracy of the model. The formula for accuracy is:


$$Accuracy=\frac{TP+TN}{TP+TN+FP+FN}$$

where, TP is true positives, TN is true negatives, FP is false positives, and FN is false negatives.

A higher accuracy indicates that the model makes correct predictions in most cases.

Precision represents the proportion of true positives among all samples predicted as positive. It primarily measures the accuracy of the model’s positive predictions. The formula for precision is:


$$Precision=\frac{TP}{TP+FP}$$

Recall, also known as sensitivity, is the proportion of true positives among all actual positive samples. A higher recall indicates fewer false negatives and a better ability of the model to identify positive samples. The formula for recall is:


$$Recall=\frac{TP}{TP+FN}$$

*F*_1_-score means the harmonic mean of precision and recall, and is used to evaluate the balance between these 2 metrics. It is suitable for datasets with class imbalance. The formula for *F*_1_-score is:


$$F1Score=2\times \frac{Precision\times Recall}{Precision＋Recall}$$

A higher *F*_1_-score indicates better performance in both precision and recall.

Specificity represents the proportion of true negatives among all actual negative samples. It measures the model’s ability to identify negative samples. The formula for specificity is:


$$Specificity=\frac{TN}{TN+FP}$$

The AUC of the receiver operating characteristic (ROC) curve is used to evaluate the overall performance of the model. The ROC curve reflects the trade-off between the true-positive rate and false-positive rate at different thresholds. A higher AUC indicates better discrimination ability of the model, balancing the true-positive rate and false-positive rate more effectively. AUC is especially useful for comparing the performance of different models, providing a global perspective on evaluation.

By comprehensively assessing these metrics, we can understand the models’ performance in various aspects, identify their strengths and weaknesses, and provide a solid basis for further optimization and improvement. This multiangle, multidimensional evaluation approach ensures a more accurate and comprehensive evaluation of the model’s performance, enhancing its reliability and effectiveness in practical applications.

### Model Interpretability

For the interpretability of ML models, we primarily used 2 widely recognized methods, LIME [28] and SHAP [29]. LIME approximates complex ML models by fitting an interpretable linear model within a local neighborhood of the target prediction point. This approach provides individualized feature importance interpretations for each instance, thus elucidating the logic behind specific model predictions. Unlike LIME, which focuses on local interpretation, SHAP quantifies the global contribution of each feature to the model prediction based on the Shapley value from game theory. The SHAP value ensures that the contribution of each feature is consistent and fair across all possible feature combinations. By calculating the SHAP value for each feature, SHAP reveals the interactions and dependencies between features and model predictions. Both methods have their respective strengths. SHAP offers a more comprehensive and in-depth interpretation, while LIME is more intuitive and flexible for instance-specific explanations. Combining the local interpretability of LIME with the global analysis of SHAP provides a more thorough and detailed interpretative support for ML models in clinical data. This combination is crucial for enhancing the transparency of the models, increasing the trust of health care professionals, and optimizing the clinical decision-making process.

### Ethical Considerations

This study conducted a secondary analysis using the publicly available Diabetes Complications Dataset [30] from the National Population Health Data Center. As this study exclusively used anonymized secondary data without direct human participant involvement, it is exempt from additional institutional ethics review. The original data collection by the National Population Health Data Center complies with the “Regulations on the Management of Human Genetic Resources of the People’s Republic of China” and relevant ethical guidelines. All data are completely deidentified with no personally identifiable information included, and formal data access approval was obtained from the data provider following their established protocols. The original informed consent permits secondary analysis of the deidentified data for research purposes.

## Results

### Feature Selection

**Figure 2.**
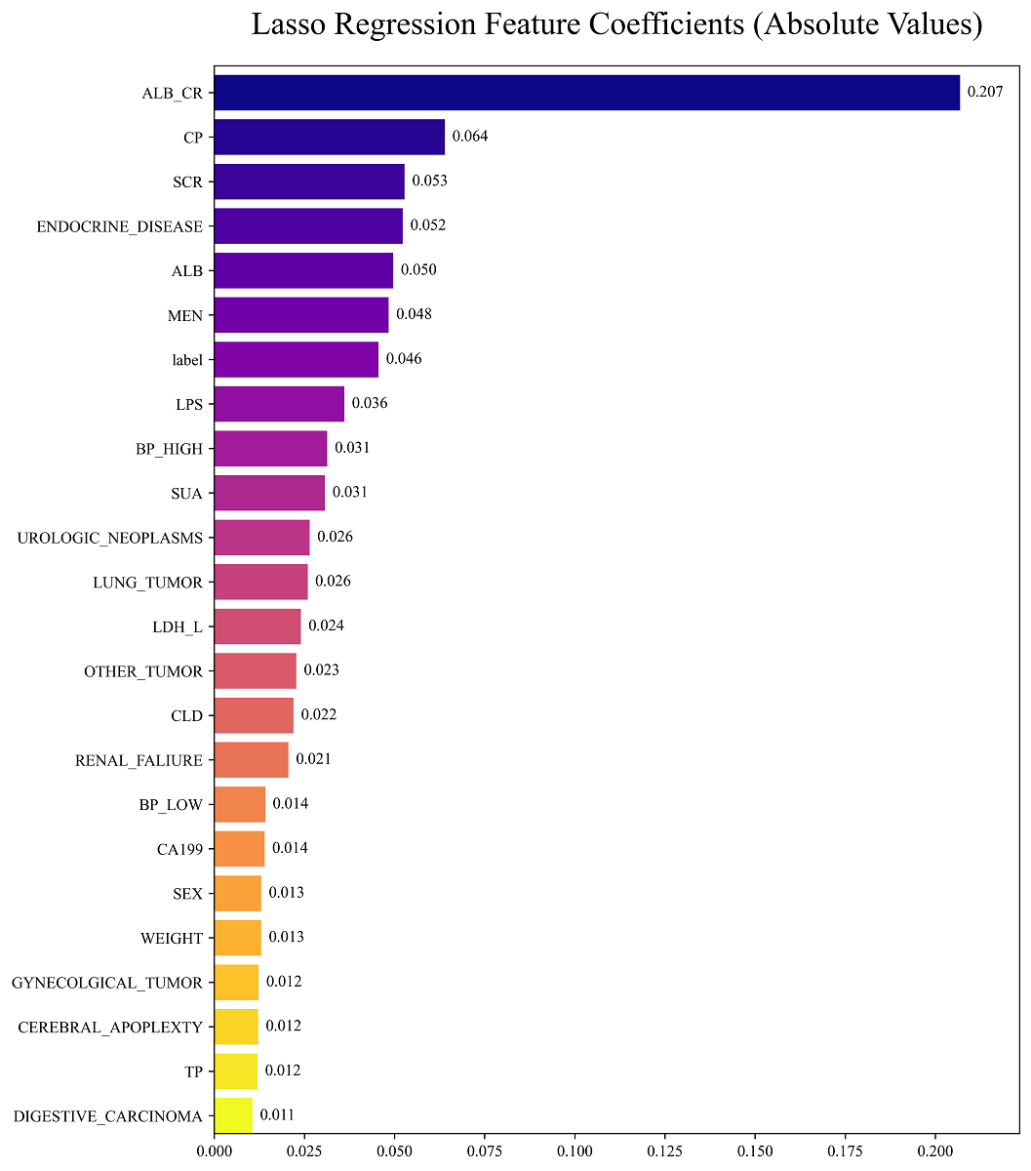
Absolute values of feature coefficients in lasso regression. The color gradient reflects the magnitude of coefficients, with darker shades indicating higher absolute values. Key features (ALB_CR, CP, SCR, ENDOCRINE_DISEASE, ALB) are highlighted as dominant contributors to model performance. ALB: albumin; ALB_CR: albumin creatinine ratio; BP_HIGH: blood pressure high; BP_low: blood pressure low; CLD: chronic liver disease; CP: C-reactive protein; LDH_L: lactate dehydrogenase L; LPS: lipoproteins; MEN: endocrine gland tumors; SCR: serum creatinine; SUA: serum uric acid; TP: total protein.

**Figure 3.**
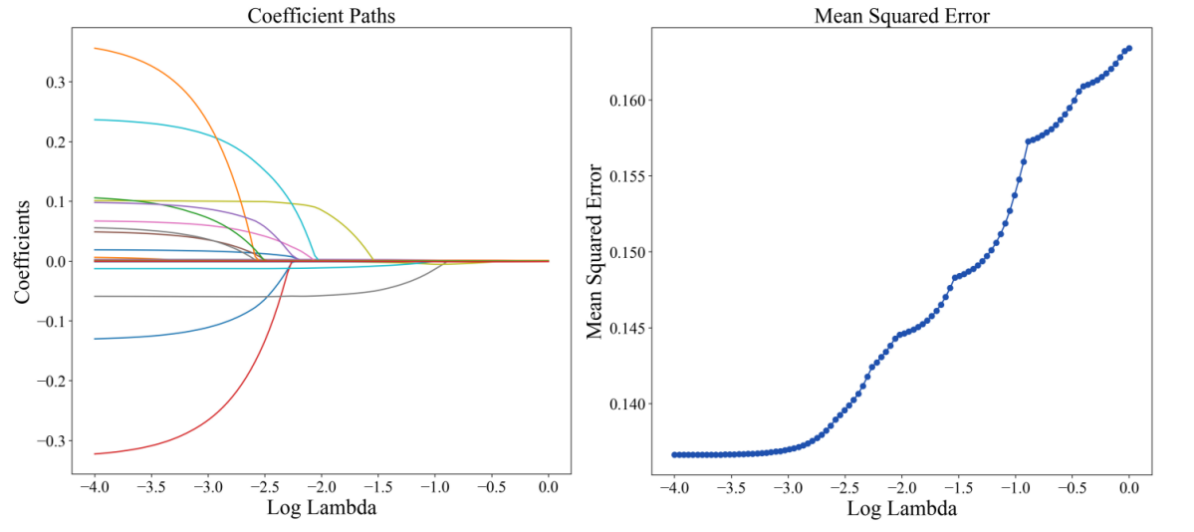
Variable selection via lasso regression. (A) Coefficient paths illustrate the trajectory of feature coefficients as a function of log(λ), demonstrating variable shrinkage and exclusion. (B) The mean-squared error curve identifies the optimal λ value at its minimum, balancing model complexity and predictive accuracy.

As illustrated in Figures2  3, lasso regression effectively filters and optimizes the variables in the model by adjusting the regularization parameter λ (Log Lambda). Each curve represents the coefficients of a feature. It can be observed that as λ increases, most coefficients gradually approach zero (Figure 3A). This phenomenon demonstrates the unique feature selection capability of lasso regression, to reduce the coefficients of unimportant features by increasing λ, and thereby simplify the model. Simultaneously, it can be seen that the mean squared error remains low when λ is small (Figure 3B), indicating that the model has a good fitting performance. However, as λ continues to increase, the mean squared error value starts to rise significantly after a relatively stable period. This trend indicates that excessive regularization will result in an overly simplified model, impairing its predictive ability. Through lasso regression, we eventually selected the 24 features, including 14 numerical features and 10 categorical ones.

Table 1 summarizes the differences in features between the DN group and the non-DN group. The statistical data reveal significant disparities between the two groups across various biochemical indicators and health conditions. Specifically, the albumin level is significantly higher in the non-DN group (mean 41.9, range 39.7-44.3) than that in the DN group (mean 38.2, range 32.4-41.4), with a *P* value of less than .001. Similar trends are observed for albumin creatinine ratio, blood pressure high, and blood pressure low. For instance, the albumin creatinine ratio is significantly lower in the non-DN group (mean 12.0, range 4.0-60.2) than that in the DN group (mean 272.7, range 79.0-472.1), with a *P* value of less than .001. Blood pressure high and blood pressure low are also lower in the non-DN group with means of 130.0 (range 120.0-142.0) and 80.0 (range 70.0-86.0) compared to the DN group with means of 142.0 (range 130.0-160.0) and 80.0 (range 75.0-90.0), respectively, both with *P* values less than .001. The levels of CA199, lactate dehydrogenase L, and lipoproteins indicate more severe renal impairment in the DN group compared to the non-DN group. For example, the CA199 level is significantly higher in the DN group (mean 16.5, range 9.5-26.3) than that in the non-DN group (mean 13.0, range 7.8-21.9), with a *P* value of less than .001. Similarly, the lactate dehydrogenase L level is higher in the DN group (mean 169.8, range 142.5-203.2) than that in the non-DN group (mean 153.2, range 135.6-176.6), with a *P* value of less than .001. Furthermore, patients with DN exhibit a higher incidence of certain clinical conditions compared to those without DN. For instance, the incidence of digestive carcinoma in the DN group is 18.33%, whereas it is 46.06% in the non-DN group, with a *P* value of less than .001. Similar significant differences are observed for other conditions such as rheumatic immunity disease, other tumors, cerebral apoplexy, and hypertension. Table 1 provides a detailed comparison of these features between the DN and non-DN groups, highlighting significant health disparities and emphasizing the need for targeted clinical interventions. Table 2 lists the variables and their corresponding abbreviations used throughout the analysis, providing a clear reference for understanding the various health indicators assessed in this study.

**Table 1.** Feature differences between diabetic nephropathy (DN) and non-DN groups.

Variable name	DN (n=444), median (IQR)	Non-DN (n=556), median (IQR)	*P* value
Albumin	38.2 (32.4-41.4)	41.9 (39.7-44.3)	<.001
Albumin_Creatinine_Ratio	272.7 (79.0-472.1)	12.0 (4.0-60.2)	<.001
Blood_Pressure_High	142.0 (130.0-160.0)	130.0 (120.0-142.0)	<.001
Blood_Pressure_Low	80.0 (75.0-90.0)	80.0 (70.0-86.0)	<.001
CA199	16.5 (9.5-26.3)	13.0 (7.8-21.9)	<.001
C-Peptide	2.4 (1.5-3.5)	2.4 (1.5-3.1)	.25
Lactate_Dehydrogenase_L	169.8 (142.5-203.2)	153.2 (135.6-176.6)	<.001
Lipoproteins	134.8 (78.6-212.7)	100.2 (48.8-163.1)	<.001
Serum_Creatinine	95.8 (68.7-161.0)	67.5 (55.2-78.7)	<.001
Serum_Uric_Acid	352.6 (292.1-416.1)	302.8 (242.6-363.2)	<.001
Total_Protein	63.3 (58.2-68.1)	67.2 (63.9-70.9)	<.001
Weight	74.0 (66.0-83.0)	72.9 (66.0-79.0)	.10
Other_Tumor, n (%)			.36
No	46 (50.55)	510 (56.11)	
Yes	45 (49.45)	399 (43.89)	
Endocrine_Disease, n (%)			<.001
No	169 (47.08)	387 (60.37)	
Yes	190 (52.92)	254 (39.63)	
Gynecological_Tumor, n (%)			>.99
No	16 (55.17)	540 (55.61)	
Yes	13 (44.83)	431 (44.39)	
Cerebral_Apoplexy, n (%)			.09
No	29 (44.62)	527 (56.36)	
Yes	36 (55.38)	408 (43.64)	
Endocrine_Gland_Tumors, n (%)			<.001
No	9 (21.95)	547 (57.04)	
Yes	32 (78.05)	412 (42.96)	
Renal_Failure, n (%)			<.001
No	0 (0.00)	556 (59.15)	
Yes	60 (100.00)	384 (40.85)	
Lung_Tumor, n (%)			.08
No	18 (75.00)	538 (55.12)	
Yes	6 (25.00)	438 (44.88)	
Urologic_Neoplasms, n (%)			.04
No	1 (12.50)	555 (59.15)	
Yes	7 (87.50)	437 (44.05)	
Gender, n (%)			.06
Male	347 (53.38)	209 (59.71)	
Female	303 (46.62)	141 (40.29)	
Chronic_Liver_Disease, n (%)			.002
No	67 (43.79)	489 (57.73)	
Yes	86 (56.21)	358 (42.27)	
Digestive_Carcinoma, n (%)			<.001
No	49 (81.67)	507 (53.94)	
Yes	11 (18.33)	433 (46.06)	
Diabetes_Disease_Type, n (%)			<.001
Type 2 diabetes	182 (36.4)	374 (74.8)	
Diabetic retinopathy	318 (63.6)	126 (25.2)	

**Table 2.** Variable names and their abbreviations.

Variable name	Abbreviation
Albumin	ALB
Albumin creatinine ratio	BU
Blood pressure high	BP_HIGH
Blood pressure low	BP_LOW
CA199	CA199
C-reactive protein	CP
Lactate dehydrogenase L	LDH_L
Lipoproteins	LPS
Serum creatinine	SCR
Serum uric acid	SUA
Total protein	TP
Weight	WEIGHT
Other tumor	OTHER_TUMOR
Other endocrine diseases	ENDOCRINE_DISEASE
Gynecological tumor	GYNECOLOGICAL_TUMOR
Cerebral apoplexy	CEREBRAL_APOPLEXTY
Endocrine gland tumors	MEN
Renal failure	RENAL_FAILURE
Lung tumor	LUNG_TUMOR
Urologic neoplasms	UROLOGIC_NEOPLASMS
Gender	SEX
Chronic liver disease	CHRONIC_LIVER_DISEASE
Digestive carcinoma	DIGESTIVE_CARCINOMA
Diabetes disease type	DIABETES_DISEASE_TYPE

### Model Performance

In this study, we used various ML algorithms to construct predictive models, including decision tree, random forest, extra trees, AdaBoost, XGBoost, and LightGBM. In order to determine the optimal model parameters, we used ten-fold cross-validation and grid search strategies, ensuring thorough and accurate model tuning. The final results, presented in Table 3, show that XGBoost and LightGBM exhibited superior performance across multiple evaluation metrics, including accuracy, precision, recall, *F*_1_-score, and specificity. In particular, XGBoost achieved the highest accuracy of 86.87%, with a precision of 88.90%, a recall of 84.40%, an *F*_1_-score of 86.44%, and a specificity of 89.12%. LightGBM followed closely with an accuracy of 86.78%, a precision of 88.72%, a recall of 84.37%, an *F*_1_-score of 86.35%, and a specificity of 88.88%. These results highlight the exceptional capability of both XGBoost and LightGBM in handling complex datasets, making them ideal for predictive modeling in this context. The other effective models, however, did not match the overall performance of XGBoost and LightGBM.

**Table 3.** Performance comparison of different machine learning models.

Model	Accuracy (%)	Precision (%)	Recall (%)	*F*_1_-score (%)	Specificity (%)
Decision tree	78.24	85.02	69.88	76.07	86.26
Random forest	85.07	88.25	80.72	84.22	89.16
Extra trees	84.26	89.06	78.32	83.16	89.98
AdaBoost^a^	83.10	85.44	79.77	82.29	86.31
XGBoost^b^	86.87	88.90	84.40	86.44	89.12
LightGBM^c^	86.78	88.72	84.37	86.35	88.88

a AdaBoost: Adaptive Boosting.

b XGBoost: Extreme Gradient Boosting.

c LightGBM: Light Gradient-Boosting Machine.

**Figure 4.**
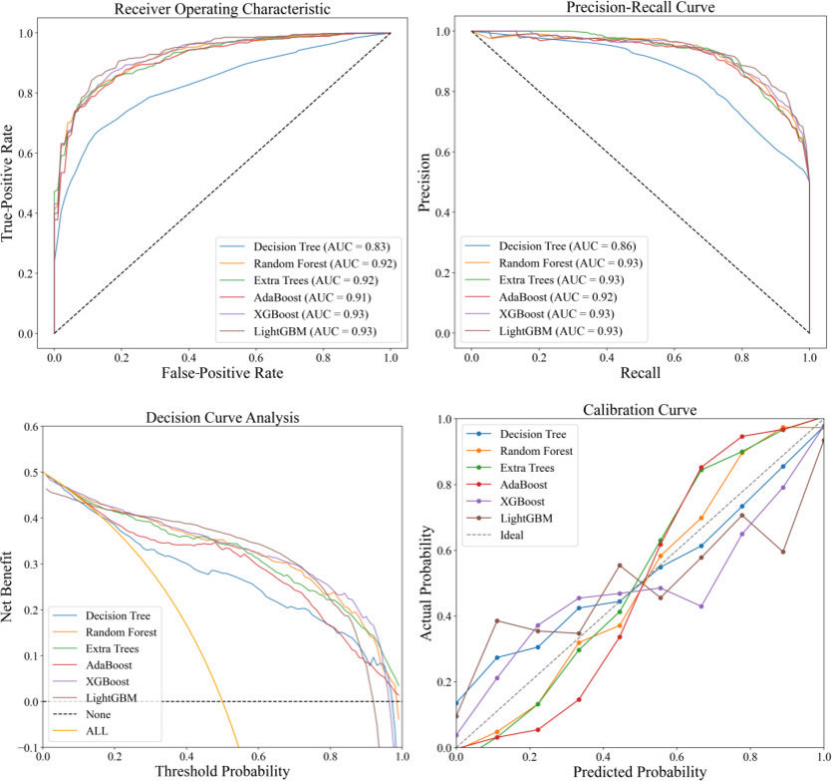
Comprehensive evaluation of model performance. (A) Receiver operating characteristic (ROC) curves demonstrate discriminative ability, with LightGBM achieving the highest AUC (0.93) [6,10]. (B) Precision-recall curves highlight performance in imbalanced datasets, where XGBoost and LightGBM maintain high precision across recall ranges. (C) Decision curve analysis reveals net clinical benefit, indicating LightGBM’s superiority over alternative models at most threshold probabilities. (D) Calibration curves assess predictive accuracy, showing LightGBM aligns closest to the ideal calibration line. AdaBoost: Adaptive Boosting; AUC: area under the curve; LightGBM: Light Gradient-Boosting Machine; XGBoost: Extreme Gradient Boosting.

As shown in Figure 4, through a comprehensive performance evaluation that included ROC curves, precision-recall curves, decision curve analysis, and calibration curves, we found that LightGBM and XGBoost performed exceptionally well. Both models demonstrated strong discriminative abilities with an AUC of 0.93, indicating their effectiveness at distinguishing between positive and negative classes. The precision-recall curves, particularly valuable for imbalanced datasets, also showed an AUC of 0.93 for both models, reflecting their capability to maintain high precision and recall, which minimizes false positives and negatives. In the decision curve analysis, XGBoost provided the highest net benefit, highlighting its robustness and wide applicability across various clinical decision thresholds. This indicates that XGBoost can be particularly useful in diverse clinical scenarios, offering reliable support for decision-making processes. The calibration curves further confirmed the reliability of the predicted probabilities from both models, showing that their predictions closely matched the actual outcomes, especially in the high probability prediction range. This means that when the models predict a high probability for an event, the prediction is likely to be accurate, which is essential for building trust in model predictions. In summary, LightGBM and XGBoost excelled across all evaluated metrics, demonstrating superior performance and robustness. Their high AUC values, significant net benefit, and accurate probability predictions collectively underscore their effectiveness in handling complex clinical datasets, making them highly suitable for predictive modeling in health care where accurate and reliable predictions are critical for effective patient management and treatment planning.

### Model Explainability

**Figure 5.**
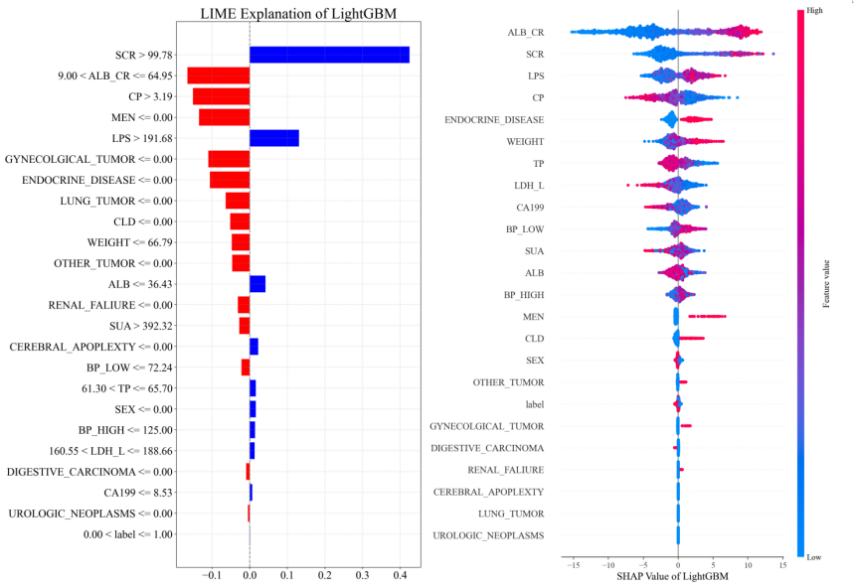
Feature importance analysis of the LightGBM model. (A) LIME explanation highlights individual feature contributions, where blue/red bars indicate positive/negative impacts on predictions. (B) SHAP values reveal global feature influence, with ALB_CR and SCR exhibiting the strongest associations (purple/blue gradient reflects feature magnitude). ALB: albumin; ALB_CR: albumin creatinine ratio; BP_HIGH: blood pressure high; BP_LOW: blood pressure low; CLD: chronic liver disease; CP: C-reactive protein; LDL_L: lactate dehydrogenase L; LightGBM: Light Gradient-Boosting Machine; LIME: Local Interpretable Model-Agnostic Explanations; LPS: lipoproteins; MEN: endocrine gland tumors; SCR: serum creatinine; SHAP: Shapley Additive Explanations; SUA: serum uric acid; TP: total protein.

In the analysis of the LIME diagram, we identified a series of key biomarkers and clinical indicators closely associated with the occurrence and progression of DN. As depicted in Figure 5A, the values next to each feature represent the contribution of that feature to the model’s prediction outcomes, denoted as the importance score. Blue indicates a positive correlation between the feature value and the model’s prediction outcomes, meaning that as the feature value increases, the likelihood of the model predicting the positive class also increases. Conversely, red indicates a negative correlation, suggesting that as the feature value increases, the likelihood of the model predicting the negative class increases. LIME technology provides an intuitive explanation of the factors influencing model predictions by constructing local surrogate models around the model’s predictions in the form of linear models, approximating the original complex model. Specifically, the level of serum creatinine was identified as a significant positive predictive factor, with a threshold exceeding 99.78 µmol/L associated with an increased risk of DN. This finding aligns with the clinical understanding that elevated creatinine levels typically indicate decreased kidney filtration capacity. Albumin levels within the range of 9.00 to 64.95 g/L were also associated with the risk of DN. As the main protein in plasma, abnormal albumin levels may indicate malnutrition or an inflammatory state, both of which can impact kidney health. The lipopolysaccharide level exceeding 191.68 mg/dL was identified as another positive predictive factor. As an inflammatory mediator, elevated lipopolysaccharide levels may be linked to the inflammatory processes in DN. The level of serum uric acid exceeding 392.32 µmol/L was also identified as a positive predictive factor. Hyperuricemia is associated with various kidney diseases and may increase the risk of DN through mechanisms such as promoting inflammation and oxidative stress. Blood pressure indicators, including diastolic blood pressure (BP_LOW) at 72.24 mmHg and systolic blood pressure (BP_HIGH) not exceeding 125.00 mmHg, were also considered to be significant in the LIME analysis. Effective blood pressure control is crucial for slowing the progression of DN. In addition, the levels of total protein within the range of 61.30 to 65.70 g/L and lactate dehydrogenase within the range of 160.55 to 188.66 U/L played a role in the model’s predictions. Abnormalities in these indicators may reflect systemic metabolic disorders or tissue damage, both of which are related to the risk of DN. The presence of other medical conditions such as gynecological tumors, endocrine diseases, and lung tumors also showed a negative correlation with the risk of DN, suggesting that these factors may have a protective or neutralizing effect in this specific dataset. Through this detailed analysis, LIME technology provided a comprehensive and intuitive explanation of the key factors influencing model predictions, thereby enhancing our understanding of the biomarkers and clinical indicators associated with DN.

Figure 5B illustrates the SHAP importance of features within the LightGBM model, identifying primary factors such as lipoprotein A, serum creatinine, C-reactive protein, albumin, and blood pressure high as crucial ones in evaluating DN. These features are pivotal across models, underscoring their integral role in disease assessment. Lipoprotein_A and other lipoproteins serve as critical lipid markers, essential for gauging the cardiovascular risks and progression of kidney disease in patients with diabetes. Abnormal lipid metabolism is intimately linked with the onset of DN, highlighting a potential need for enhanced lipid management strategies to thwart disease advancement. Furthermore, Serum_Creatinine and Blood_Urea nitrogen are paramount biochemical indicators for renal function assessment. Elevated concentrations of these markers generally denote diminished kidney filtration ability, serving as significant indicators of DN progression. Regular monitoring of these parameters is vital for the early detection of renal impairment and timely medical intervention. Albumin serves as a sensitive biomarker for early DN. The abnormal rate of urinary albumin excretion reflects the extent of renal damage. Early detection and continual monitoring of urinary albumin levels enable physicians to assess disease progression and the efficacy of their treatment protocols, thereby facilitating the development of customized therapeutic strategies aimed at decelerating disease progression. Hypertension significantly accelerates DN development; thus, effective blood pressure management is imperative for delaying or preventing disease progression. Optimal control of hypertension not only preserves renal function but also mitigates the risk of cardiovascular incidents. The collective significance of these features underscores their pivotal role in DN assessment. Through a comprehensive analysis of these factors, physicians can obtain a deeper understanding of the disease’s complexity, and develop more effective monitoring and treatment modalities that enhance patients’ management and prognosis.

**Figure 6.**
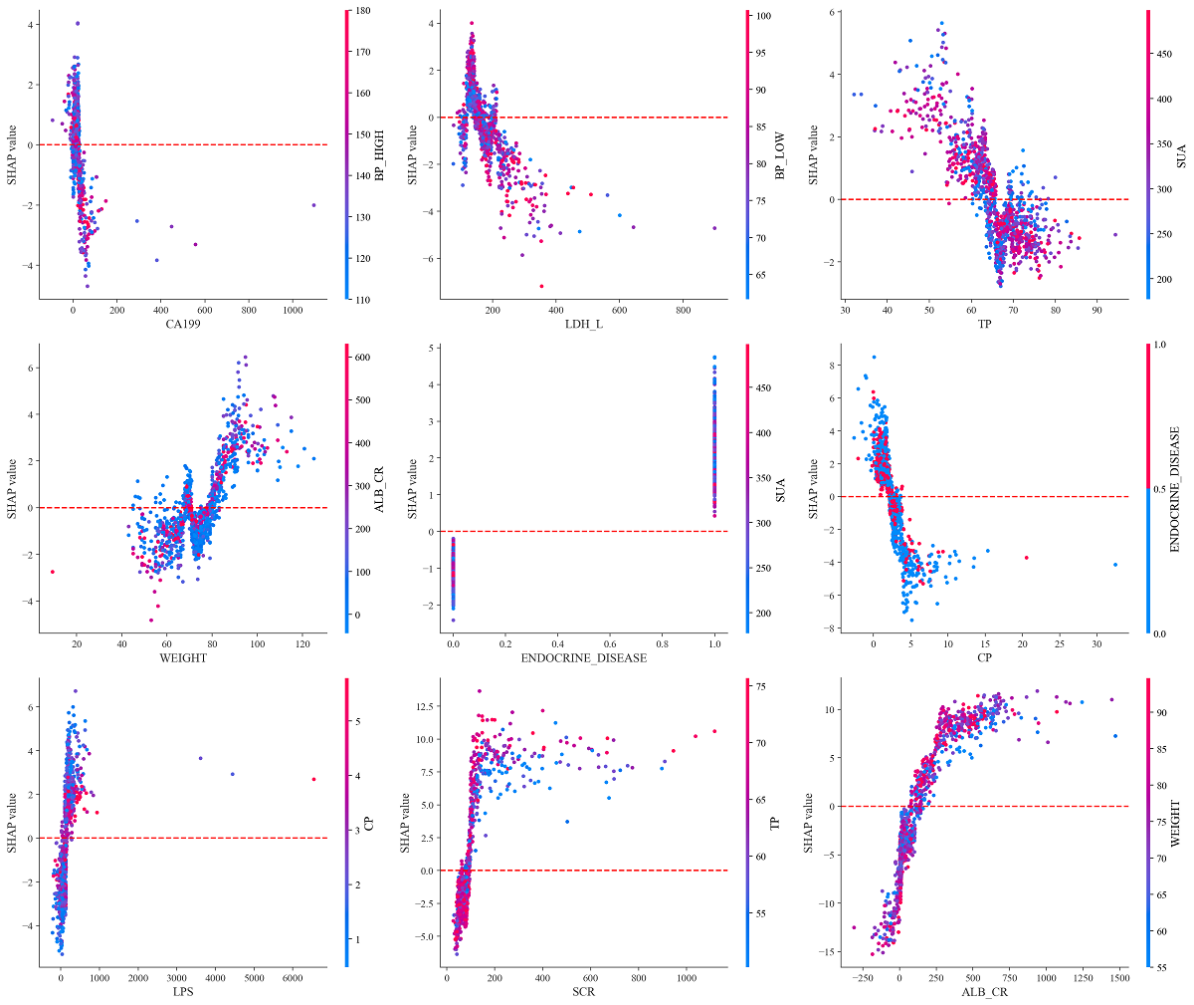
SHAP value distributions for key biochemical indicators in nephropathy risk prediction. Each subplot illustrates the relationship between feature values (x-axis) and SHAP values (y-axis), with color gradients indicating feature magnitude. Red dashed lines denote baseline thresholds, where features above/below these thresholds significantly influence model predictions. Notably, ALB_CR and SCR exhibit the strongest associations with nephropathy risk due to their wide SHAP value ranges and distinct clustering patterns. Detailed subfigures are available in MultimediaAppendices 1^9^. ALB_CR: albumin creatinine ratio; BP_HIGH: blood pressure high; CP: C-reactive protein; LDL_L: lactate dehydrogenase L; SCR: serum creatinine; SHAP: Shapley Additive Explanations; SUA: serum uric acid; TP: total protein.

Figure 6 delineates the correlation between various biochemical indicators and their SHAP values in interpretable ML models designed for predicting and evaluating the risk of DN. An in-depth analysis of these biochemical markers furnishes critical scientific insights into their influence on DN risk predictions, thereby informing clinical intervention strategies. The analysis reveals a pronounced negative correlation between the levels of blood urea nitrogen (blood urea) and the model’s predictive accuracy. As blood urea nitrogen levels increase, the associated SHAP values decline markedly, signifying an augmented risk of DN. This trend underscores the prognostic importance of blood urea nitrogen in the early detection of renal dysfunction, establishing it as an essential indicator for kidney disease risk assessments. Similarly, fluctuations in albumin levels are critical for forecasting DN. The presented data indicate that rising albumin levels correlate with decreasing SHAP values, underscoring albumin’s predictive utility, particularly in detecting microalbuminuria—an early manifestation of kidney damage. Elevated levels of C-reactive protein, a marker of systemic inflammation, also correspond with reduced SHAP values, suggesting that high concentrations of this protein are linked to an increased risk of DN. This relationship may stem from chronic inflammatory states that foster the progression of DN. The findings concerning serum creatinine demonstrate that increased creatinine levels are indicative of reduced kidney filtration capabilities, mirrored by a decline in SHAP values. This reinforces the value of serum creatinine as a crucial indicator of renal insufficiency and a metric for assessing the risk of DN. The analysis also highlights the significant negative impact of elevated lipoprotein markers on the model’s predictions, emphasizing the imperative role of lipid management in the prevention and treatment of DN. The perturbations in these indicators not only relate to cardiovascular diseases but also pose a substantial risk factor for DN. Furthermore, the notable SHAP values for endocrine disease and diabetes type underscore the critical role of these conditions in assessing DN risk, indicating that they are significant contributors to the disease’s development. By synthesizing these data, we can enhance our comprehension of the risk factors associated with DN. This integration aids medical professionals in more accurately identifying patients at high risk, thereby facilitating the development of more effective treatment strategies and management practices to improve the renal health of patients with diabetes.

**Figure 7.**
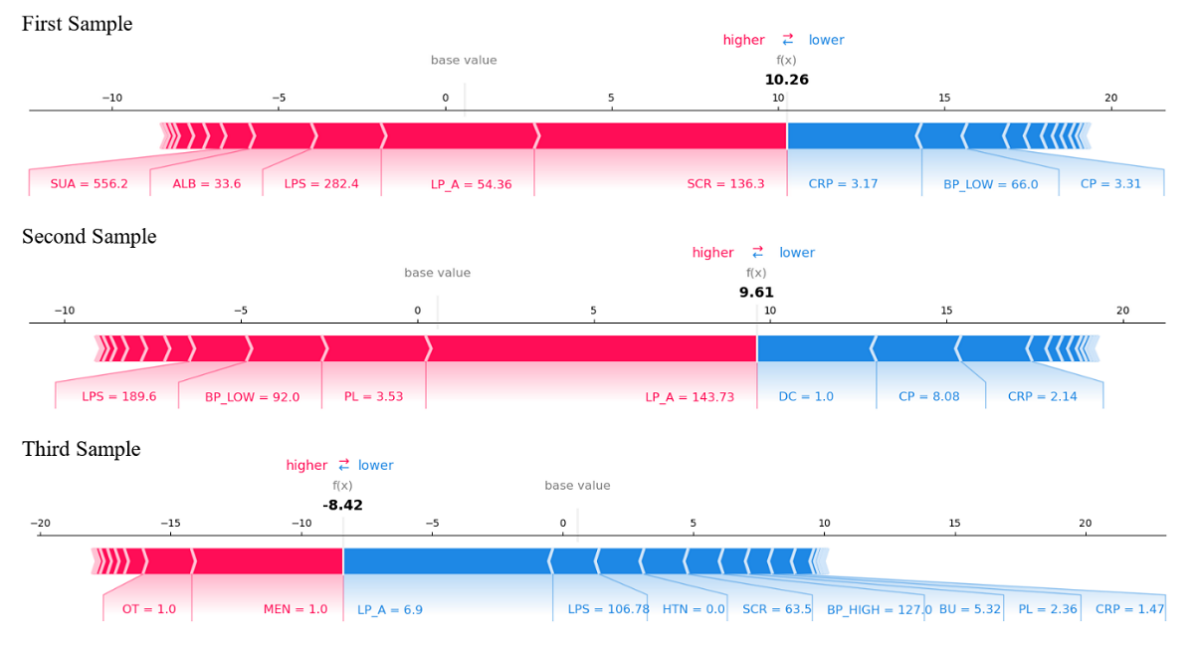
Comparative analysis of Shapley Additive Explanations (SHAP) values across three patient samples for diabetic nephropathy prediction. Each waterfall plot illustrates individual feature contributions to the final prediction score, where red/blue segments indicate positive/negative impacts on diabetic nephropathy risk. The base value represents the average model output without feature effects, while the final f(x) reflects personalized risk assessment. Notably, SUA and ALB exhibit consistent positive associations in high-risk samples (first/second), whereas MEN and HTN dominate negative contributions in low-risk cases (third). ALB: albumin; BP_HIGH: blood pressure high; BP_LOW: blood pressure low; BU: albumin creatinine ratio; CP: C-peptide; CRP: C-reactive protein; LP_A: lysophosphatidic acid; LPS: lipoproteins; DC: digestive carcinoma; HTN: hypertension; LPS: lipoproteins; MEN: endocrine gland tumors; OT: other tumor; PL: phospholipid; SCR: serum creatinine; SUA: serum uric acid.

For the purpose of evaluating the predictive models for DN, the SHAP value plots for three distinct samples (Figure 7) offer a nuanced understanding of the contribution of various biochemical and physiological indicators to disease risks prediction. These comprehensive data enable insights into how different indicators, either individually or synergistically, influence disease risk assessment, thereby furnishing a scientific foundation for clinical decision-making. The analysis of the first sample elucidates that elevated levels of serum creatinine and C-reactive protein markedly enhance disease risks. Serum creatinine, a key marker of renal function impairment, signals a significant reduction in kidney function, which is particularly crucial in DN where renal lesions often correlate with prolonged suboptimal diabetes control. C-reactive protein, indicative of the body’s inflammatory response, suggests the presence of an inflammatory state that is instrumental in accelerating renal damage. Furthermore, hypertension is a significant risk factor for DN, and the notably increased low blood pressure might suggest inadequate blood pressure management in this sample, exacerbating the renal burden. Conversely, higher levels of serum uric acid and albumin are generally viewed as protective in the first sample. Although elevated serum uric acid levels are linked to other conditions like gout, they may indicate preserved renal excretion function to a degree. Albumin, a crucial component of plasma proteins, with maintained levels suggests a favorable nutritional state and some preservation of kidney filtration function. The analysis of the second sample, with its positive prediction value indicating disease absence, highlights the critical role of lipoprotein A in increasing disease risks. Lipoprotein A, an independent risk factor for cardiovascular diseases, underscores a possible link between cardiovascular and renal health in the context of DN. Moreover, an elevated level of serum lipase (lipopolysaccharides) contributes to lowering disease risks in this sample, possibly due to its role in modulating immune responses and inflammation. The third sample’s negative prediction value signals a heightened disease risk. In this context, increased serum creatinine and high blood pressure significantly elevate disease risks, emphasizing the importance of vigilant monitoring and control of these indicators. Although blood urea contributes less significantly to the prediction value, its elevation is typically associated with renal insufficiency and warrants clinical attention. Through this meticulous SHAP value–based analysis, we unveil not only the specific contributions of each biochemical indicator to disease risks but also the intricate interplay and connections among these indicators. This profound understanding provides invaluable information for medical professionals in the early diagnosis, risk assessment, and therapeutic decision-making of DN, facilitating personalized medicine and enhancing treatment outcomes of patients.

## Discussion

### Principal Findings

In this study, we focused on developing a predictive model for DN risks and conducted an extensive feature analysis, aiming to identify patients at risk of progressing to severe kidney disease in a clinical setting. To achieve this goal, we thoroughly analyzed a range of biomarkers and clinical indicators and evaluated several popular ML algorithms. Among these various algorithms tested, we found that the gradient-boosting–based algorithms, XGBoost and LightGBM, performed exceptionally well on our dataset. The models not only demonstrated significant advantages in training speed and handling large datasets but also exhibited superior predictive performance across key metrics such as accuracy, precision, recall, and AUC, showcasing their outstanding predictive capabilities. Our study also found that serum creatinine, the albumin-creatinine ratio (ACR), abnormal levels of lipoproteins, C-peptide, and hypertension were the most relevant factors associated with DN, aligning with findings from clinical research on the key risk factors for DN progression.

### Comparison With Prior Work

Serum creatinine is a key biomarker for evaluating DN and core indicator of kidney function, and its elevated levels are often closely associated with kidney damage. In patients with diabetes, even a slight increase in serum creatinine levels is sufficient to indicate early kidney function impairment [31]. As serum creatinine levels rise, the risk of kidney damage also increases; thus, it is recommended to closely monitor serum creatinine levels in diabetes management [32]. Serum creatinine levels have independent predictive value in the progression of DN. Regular monitoring of serum creatinine levels helps the early identification of potential kidney disease risks and the taking of appropriate preventive measures [33]. By optimizing medical management for patients with diabetes and regularly assessing serum creatinine levels, clinicians can detect and address kidney function damage earlier, thereby reducing the progression risk of DN.

The ACR is an important method for detecting microalbuminuria, used to evaluate early changes in kidney disease in patients with diabetes. An increase in ACR is closely related to the progression of DN, and even a slight elevation in ACR should be considered as a warning signal for the development of DN [34]. Elevated ACR is an independent predictor for the progression of DN, and regular monitoring of ACR levels helps the early identification of potential kidney disease risks, aiding clinicians in assessing disease progression and treatment response [35]. Moreover, a reduction in ACR is associated with a slowdown in the progression of DN and a decrease in cardiovascular event rates. By optimizing medical management for patients with diabetes and regularly monitoring ACR levels, clinicians can detect and address kidney function damage earlier, thus reducing the risk of progression of DN [36].

Abnormal levels of lipoproteins, particularly elevated low-density lipoprotein (LDL) and very low–density lipoprotein (VLDL), are significant risk factors in the development of diabetic kidney disease. These lipid particles damage vascular endothelium, thereby accelerating the progression of diabetic kidney disease. Research indicates that changes in the size and number of LDL particles in patients with diabetes are significantly correlated with renal function impairment [37]. Elevated LDL levels not only lead to atherosclerosis but also directly harm renal function by promoting inflammation and oxidative stress. This damage mechanism is primarily manifested by the thickening of the glomerular basement membrane and glomerulosclerosis, which may ultimately result in renal failure [38]. Similarly, elevated VLDL levels are closely associated with the progression of kidney disease. VLDL carries a large amount of triglycerides and can be absorbed by renal tubular cells, causing cytotoxic reactions that further exacerbate tubular damage and accelerate the progression of kidney disease [39]. Even after other comorbid factors are controlled, high LDL levels remain significantly associated with the progression of kidney disease. Multiple studies have also shown that high LDL levels are closely related to increased urinary protein excretion and decreased renal function [40,41]. Therefore, managing lipoprotein levels is crucial to slowing the progression of diabetic kidney disease. Dietary adjustments, medication treatments such as statins, and lifestyle changes can effectively lower LDL and VLDL levels, thus protecting renal function.

C-peptide is an important marker for evaluating β-cell function in the pancreas, with its levels closely associated with metabolic control in diabetes. Low C-peptide levels are linked to an increased risk of DN, possibly due to metabolic disorders caused by insufficient insulin secretion. In contrast, high C-peptide levels are associated with a reduced risk of renal function deterioration in patients with type 2 diabetes. Research indicates that patients with higher C-peptide levels would experience slower declines in renal function and less increase in albuminuria [42]. Moreover, C-peptide shows potential in the treatment of DN. C-peptide replacement therapy can reduce glomerular hyperfiltration, decrease albumin excretion, and prevent glomerular and renal hypertrophy [43]. Supporting this view, another study demonstrates that C-peptide at physiological concentrations can improve glomerular filtration rate and increase renal plasma flow, and effect further validation in clinical trials [44]. C-peptide levels play a crucial role in the progression and management of DN. Monitoring and potentially modulating these levels through therapeutic interventions can provide significant benefits for the prevention and treatment of DN.

Hypertension is a major risk factor for the development and progression of DN. Persistent hypertension can lead to structural and functional changes in the renal vasculature, accelerating the decline in renal function. Patients with hypertension experience faster progression of kidney damage and thus require earlier intervention to prevent further deterioration of renal function [45]. In the context of hypertension, a sustained high pressure load on the renal blood vessels results in the thickening and hardening of the vessel walls. These structural changes weaken the kidney’s filtration function and accelerate glomerulosclerosis and renal fibrosis [34]. Effective hypertension management typically includes the use of angiotensin-converting enzyme inhibitors and angiotensin receptor blockers, which not only lower blood pressure but also provide renal protection [46]. On the other hand, although hypotension is less common in patients with diabetes, it can have negative effects on renal function in certain situations. In severe circulatory failure or shock, hypotension can lead to inadequate renal perfusion, reducing blood flow to the kidneys and exacerbating renal damage. In clinical practice, the management strategies for hypertension and hypotension need to be individualized. For patients with hypertension, the goal is to reduce blood pressure to recommended levels while avoiding excessive hypotension. For patients at risk of hypotension, particularly older adults, close monitoring of blood pressure fluctuations is essential to prevent inadequate renal perfusion. Overall, blood pressure management plays a crucial role in the prevention and treatment of DN. Appropriate pharmacological treatment and lifestyle interventions can control blood pressure effectively, protect renal function, and delay the progression of DN.

### Limitations

This study has made notable progress in predicting DN; however, certain limitations remain. First, the data used in the study were sourced from a single medical institution. Although the model performed well on this dataset, validation with data from other institutions is needed to assess its effectiveness and generalizability. Additionally, the model has not yet undergone comprehensive clinical validation, and some features selected by the algorithm may lack diagnostic significance, while commonly used clinical indicators might not have been included.

### Future Directions

Future research should focus on the following improvements: collecting data from multiple institutions to validate the model’s robustness, strengthening collaboration with clinical experts to optimize feature selection based on clinical knowledge, and investigating potential nonlinear relationships among features. Developing a user-friendly online platform and a simplified scoring system would make the model more intuitive and accessible for clinical use. Furthermore, incorporating multimodal data, such as imaging and genomic information, could enhance the model’s predictive performance and clinical value, providing robust support for the early diagnosis and personalized treatment of DN.

### Conclusion

In this study, we developed a robust predictive model for DN using various ML techniques. Among the models tested, XGBoost and LightGBM demonstrated superior performance, achieving notable metrics across other performance indicators. The integration of XML techniques, such as LIME and SHAP, provided valuable insights into the contribution of individual features, enhancing the model’s transparency and interpretability, which is crucial for clinical application. Our analysis identified several significant risk factors for DN, including serum creatinine, C-peptide, albumin, and lipoproteins. These findings are well-supported by extensive literature, reinforcing the reliability and relevance of our predictive model. The ability to accurately predict DN and understand the underlying risk factors allows for early intervention and personalized treatment strategies, ultimately improving patients’ outcomes and optimizing health care resource usage.

## Supplementary material

10.2196/64979 Multimedia Appendix 1 Shapley Additive Explanations (SHAP) scatter plot for CA199.

10.2196/64979 Multimedia Appendix 2 Shapley Additive Explanations (SHAP) scatter plot for lactate dehydrogenase L.

10.2196/64979 Multimedia Appendix 3 Shapley Additive Explanations (SHAP) scatter plot for total protein.

10.2196/64979 Multimedia Appendix 4 Shapley Additive Explanations (SHAP) scatter plot for weight.

10.2196/64979 Multimedia Appendix 5 Shapley Additive Explanations (SHAP) scatter plot for endocrine disease.

10.2196/64979 Multimedia Appendix 6 Shapley Additive Explanations (SHAP) scatter plot for C-reactive protein.

10.2196/64979 Multimedia Appendix 7 Shapley Additive Explanations (SHAP) scatter plot for lipoproteins.

10.2196/64979 Multimedia Appendix 8 Shapley Additive Explanations (SHAP) scatter plot for serum creatinine.

10.2196/64979 Multimedia Appendix 9 Shapley Additive Explanations (SHAP) scatter plot for albumin creatinine ratio.

10.2196/64979 Multimedia Appendix 10 Diabetes Complications Dataset.

## Abbreviations

ACR: albumin-creatinine ratio
AdaBoost: Adaptive Boosting
AI: artificial intelligence
AUC: area under the curve
DN: diabetic nephropathy
lasso: least absolute shrinkage and selection operator
LDL: low-density lipoprotein
LightGBM: Light Gradient-Boosting Machine
LIME: Local Interpretable Model-Agnostic Explanations
ML: machine learning
PDP: partial dependence plot
ROC: receiver operating characteristic
SHAP: Shapley Additive Explanations
SMOTE: Synthetic Minority Oversampling Technique
VLDL: very low–density lipoprotein
XGBoost: Extreme Gradient Boosting
XML: explainable machine learning

## References

[1] Agarwal R (2021). Pathogenesis of diabetic nephropathy. Compendia.

[2] Umanath K, Lewis JB (2018). Update on diabetic nephropathy: core curriculum 2018. Am J Kidney Dis.

[3] Zhang L, Wang Z, Zhang X (2022). Alterations of the gut microbiota in patients with diabetic nephropathy. Microbiol Spectr.

[4] Pereira PR, Carrageta DF, Oliveira PF, Rodrigues A, Alves MG, Monteiro MP (2022). Metabolomics as a tool for the early diagnosis and prognosis of diabetic kidney disease. Med Res Rev.

[5] Naaman SC, Bakris GL (2023). Diabetic nephropathy: update on pillars of therapy slowing progression. Diabetes Care.

[6] Richens JG, Lee CM, Johri S (2020). Improving the accuracy of medical diagnosis with causal machine learning. Nat Commun.

[7] Rajula HSR, Verlato G, Manchia M, Antonucci N, Fanos V (2020). Comparison of conventional statistical methods with machine learning in medicine: diagnosis, drug development, and treatment. Med Bogota Colomb.

[8] Dong J, Feng T, Thapa-Chhetry B (2021). Machine learning model for early prediction of acute kidney injury (AKI) in pediatric critical care. Crit Care.

[9] Lee YW, Choi JW, Shin EH (2021). Machine learning model for predicting malaria using clinical information. Comput Biol Med.

[10] Rahimi M, Akbari A, Asadi F, Emami H (2023). Cervical cancer survival prediction by machine learning algorithms: a systematic review. BMC Cancer.

[11] Farah L, Murris JM, Borget I, Guilloux A, Martelli NM, Katsahian SIM (2023). Assessment of performance, interpretability, and explainability in artificial intelligence-based health technologies: what healthcare stakeholders need to know. Mayo Clin Proc Digit Health.

[12] Li Z, Bouazizi M, Ohtsuki T, Ishii M, Nakahara E (2024). Toward building trust in machine learning models: quantifying the explainability by SHAP and references to human strategy. IEEE Access.

[13] El Shawi R, Sherif Y, Al-Mallah M, Sakr S Interpretability in healthcare a comparative study of local machine learning interpretability techniques.

[14] Chadaga K, Prabhu S, Sampathila N (2024). Explainable artificial intelligence approaches for COVID-19 prognosis prediction using clinical markers. Sci Rep.

[15] Khanna VV, Chadaga K, Sampathila N (2023). A decision support system for osteoporosis risk prediction using machine learning and explainable artificial intelligence. Heliyon.

[16] Guan C, Ma F, Chang S, Zhang J (2023). Interpretable machine learning models for predicting venous thromboembolism in the intensive care unit: an analysis based on data from 207 centers. Crit Care.

[17] Zhong Y, Jatav A, Afrin K, Shivaram T, Bukkapatnam STS (2023). Enhanced SpO_2_ estimation using explainable machine learning and neck photoplethysmography. Artif Intell Med.

[18] Suh B, Yu H, Kim H (2023). Interpretable deep-learning approaches for osteoporosis risk screening and individualized feature analysis using large population-based data: model development and performance evaluation. J Med Internet Res.

[19] Bernard D, Doumard E, Ader I (2023). Explainable machine learning framework to predict personalized physiological aging. Aging Cell.

[20] Zhang J, Cui X, Yang C (2023). A deep learning‐based interpretable decision tool for predicting high risk of chemotherapy‐induced nausea and vomiting in cancer patients prescribed highly emetogenic chemotherapy. Cancer Med.

[21] Tibshirani R (1996). Regression shrinkage and selection via the lasso. J R Stat Soc Series B Stat Methodol.

[22] Quinlan JR (1986). Induction of decision trees. Mach Learn.

[23] Breiman L (2001). Random forests. Mach Learn.

[24] Geurts P, Ernst D, Wehenkel L (2006). Extremely randomized trees. Mach Learn.

[25] Freund Y, Schapire RE (1997). A decision-theoretic generalization of on-line learning and an application to boosting. J Comput Syst Sci.

[26] Chen T, Guestrin C XGBoost: a scalable tree boosting system.

[27] Ke G, Meng Q, Finley T, Guyon I, Von Luxburg U, Bengio S, Wallach H, Fergus R, Vishwanathan S, Garnett R (2017). Advances in Neural Information Processing Systems 30 (NIPS 2017).

[28] Ribeiro MT, Singh S, Guestrin C (2016). “Why Should I Trust You?”: explaining the predictions of any classifier. arXiv.

[29] Lundberg S, Lee SI (2017). A unified approach to interpreting model predictions.

[30] Diabetes Complications Data Set. Population Health Data Archive.

[31] Narva AS, Bilous RW (2015). Laboratory assessment of diabetic kidney disease. Diabetes Spectr.

[32] Neumiller JJ, Alicic RZ, Tuttle KR (2024). Optimization of guideline-directed medical therapies in patients with diabetes and chronic kidney disease. Clin Kidney J.

[33] American Diabetes Association Professional Practice Committee (2023). 11. Chronic kidney disease and risk management: standards of care in diabetes—2024. Diabetes Care.

[34] Davis KN, Hines AE, Schaefer MC, Naseman KW (2022). Protecting the kidneys: update on therapies to treat diabetic nephropathy. Clin Diabetes.

[35] Li B, Wang J, Ye W (2024). A meta-analysis of urinary transferrin for early diagnosis of diabetic nephropathy. Lab Med.

[36] Sulaiman MK (2019). Diabetic nephropathy: recent advances in pathophysiology and challenges in dietary management. Diabetol Metab Syndr.

[37] Barbagallo CM, Cefalù AB, Giammanco A (2021). Lipoprotein abnormalities in chronic kidney disease and renal transplantation. Life (Basel).

[38] Gupta A, Gupta R (2023). Current understanding of diabetic dyslipidemia: a review. J Indian Inst Sci.

[39] Chen J, Fang Z, Luo Q (2024). Unlocking the mysteries of VLDL: exploring its production, intracellular trafficking, and metabolism as therapeutic targets. Lipids Health Dis.

[40] Weldegiorgis M, Woodward M (2022). Elevated triglycerides and reduced high-density lipoprotein cholesterol are independently associated with the onset of advanced chronic kidney disease: a cohort study of 911,360 individuals from the United Kingdom. BMC Nephrol.

[41] Bauer F, Seibert FS, Rohn B, Babel N, Westhoff TH (2021). Estimation of LDL cholesterol in chronic kidney disease. Eur J Prev Cardiol.

[42] Yang Q, Liu Y, Peng J (2023). High levels of serum C-peptide are associated with a decreased risk for incident renal progression in patients with type 2 diabetes: a retrospective cohort study. BMJ Open Diabetes Res Care.

[43] Wahren J, Ekberg K, Samnegård B, Johansson BL (2001). C-peptide: a new potential in the treatment of diabetic nephropathy. Curr Diab Rep.

[44] Hills CE, Brunskill NJ, Squires PE (2010). C-peptide as a therapeutic tool in diabetic nephropathy. Am J Nephrol.

[45] Colbert GB, Elrggal ME, Gaddy A, Madariaga HM, Lerma EV (2023). Management of hypertension in diabetic kidney disease. J Clin Med.

[46] Steigerwalt S (2008). Management of hypertension in diabetic patients with chronic kidney disease. Diabetes Spectr.

