# Supplementary figures and images for: Interpretable Machine Learning Model for Predicting and Assessing the Risk of Diabetic Nephropathy: Prediction Model Study
Source: JMIR Med Inform. 2025 Oct 22;13:e64979. doi: 10.2196/64979 (PMC12543291; doi:10.2196/64979)

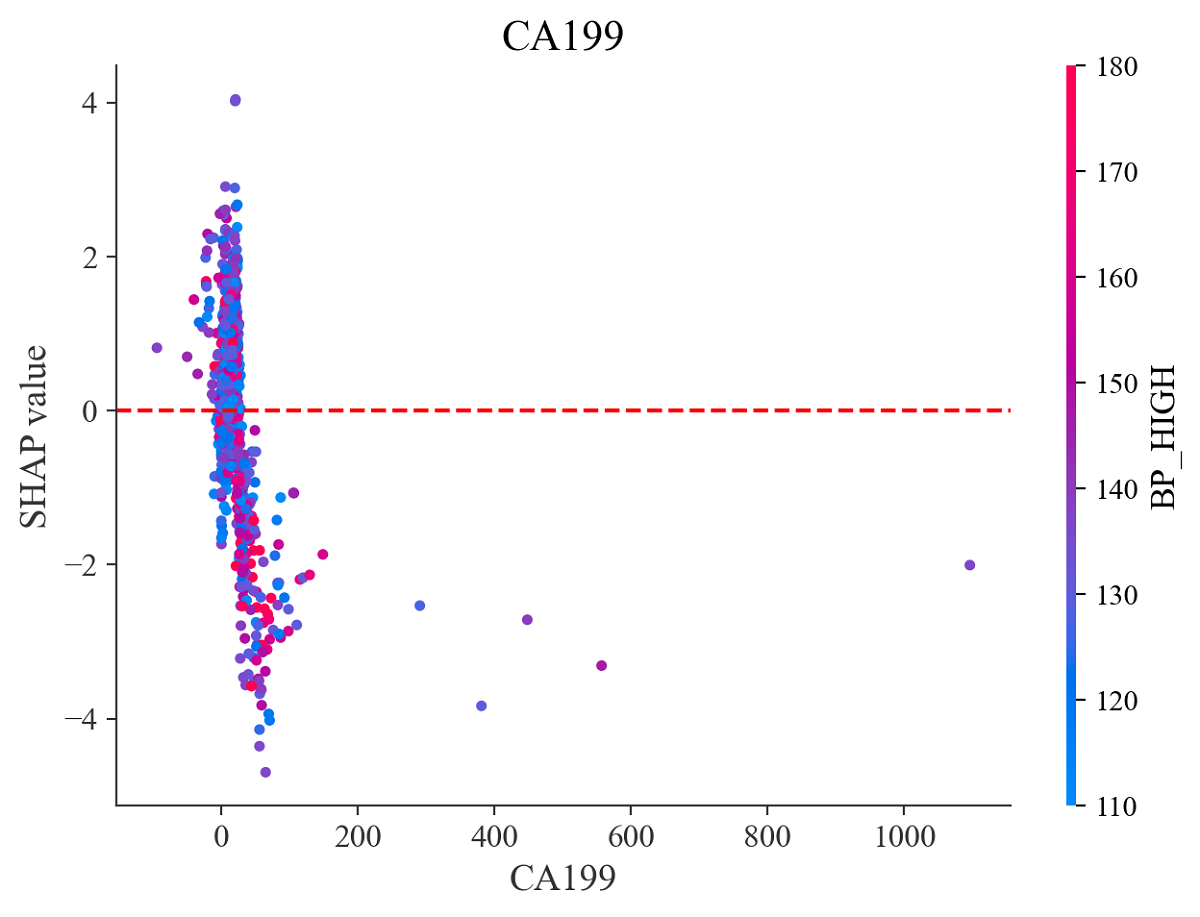

Supplement: Multimedia Appendix 1 [file medinform-v13-e64979-s001.png]

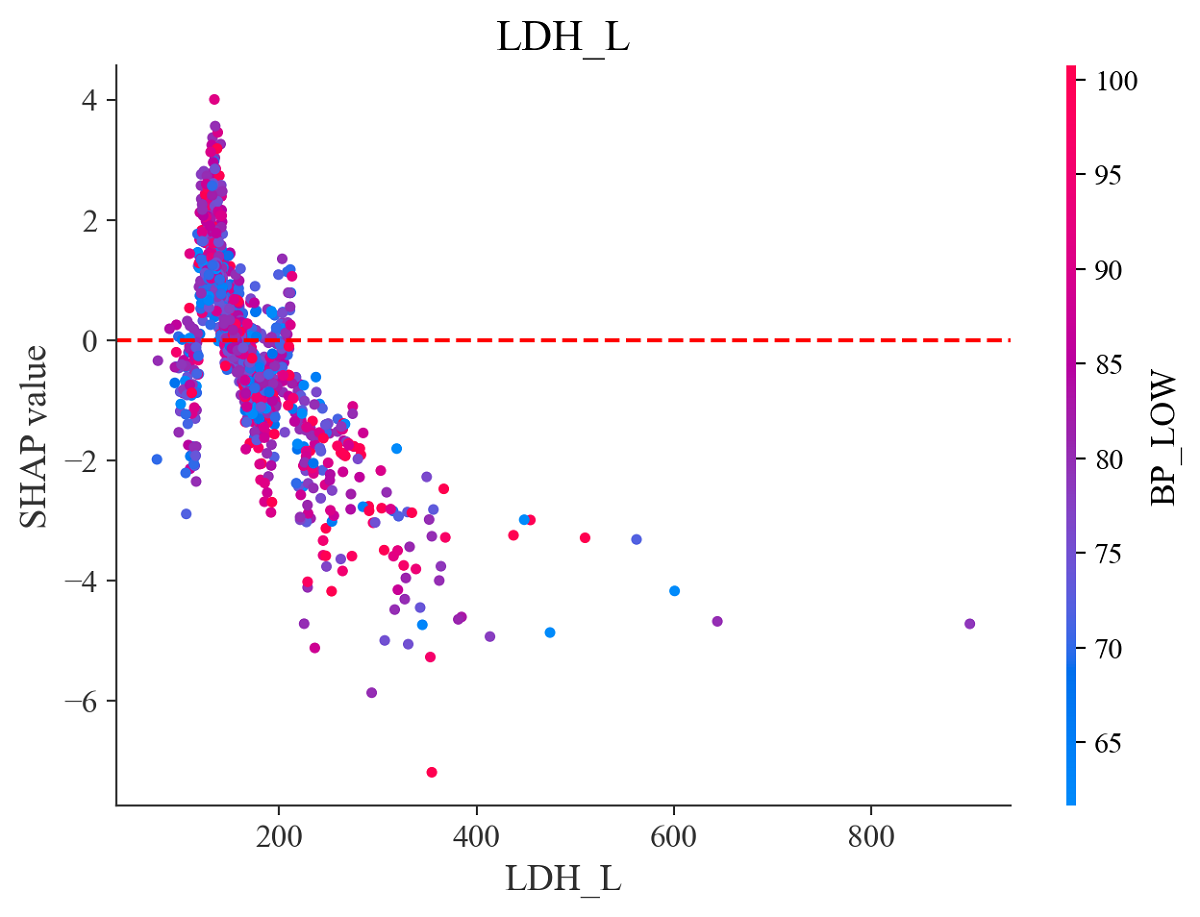

Supplement: Multimedia Appendix 2 [file medinform-v13-e64979-s002.png]

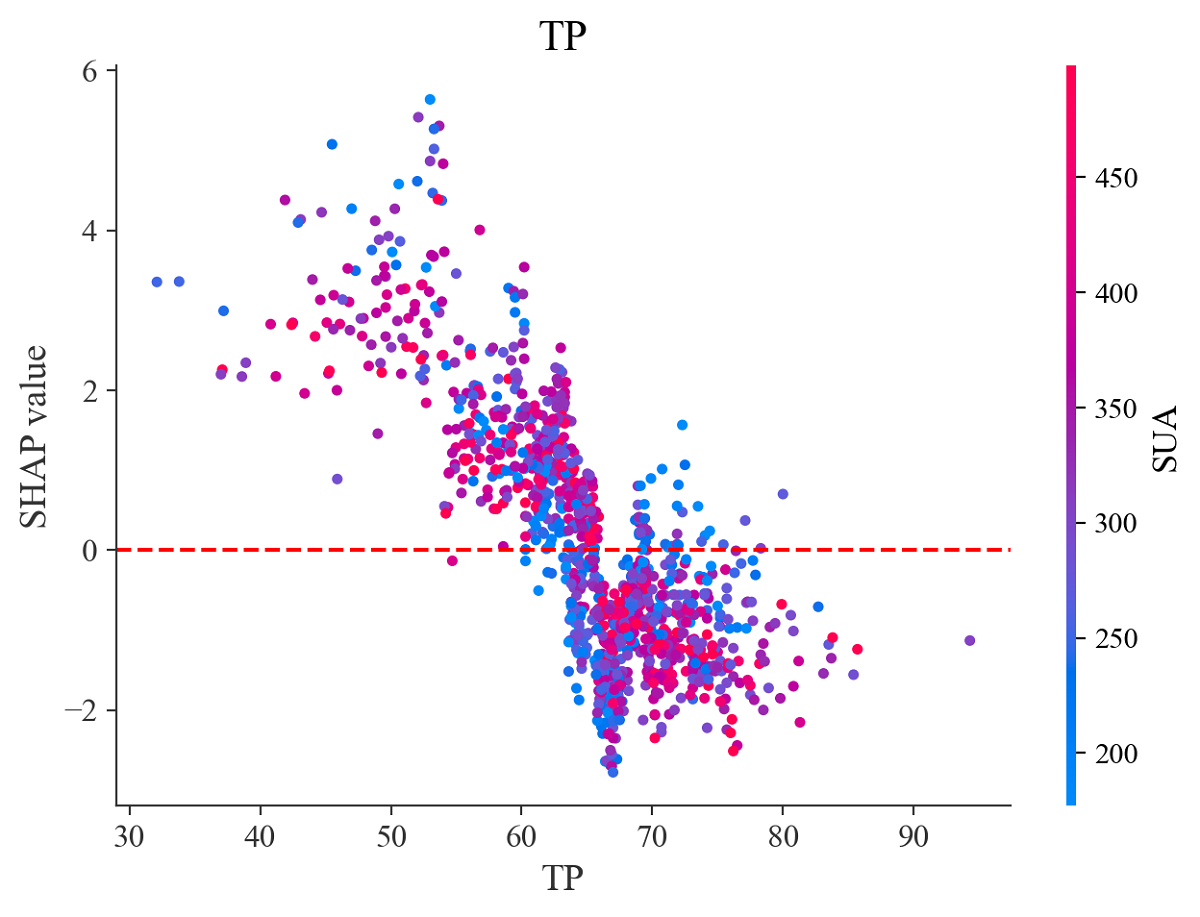

Supplement: Multimedia Appendix 3 [file medinform-v13-e64979-s003.png]

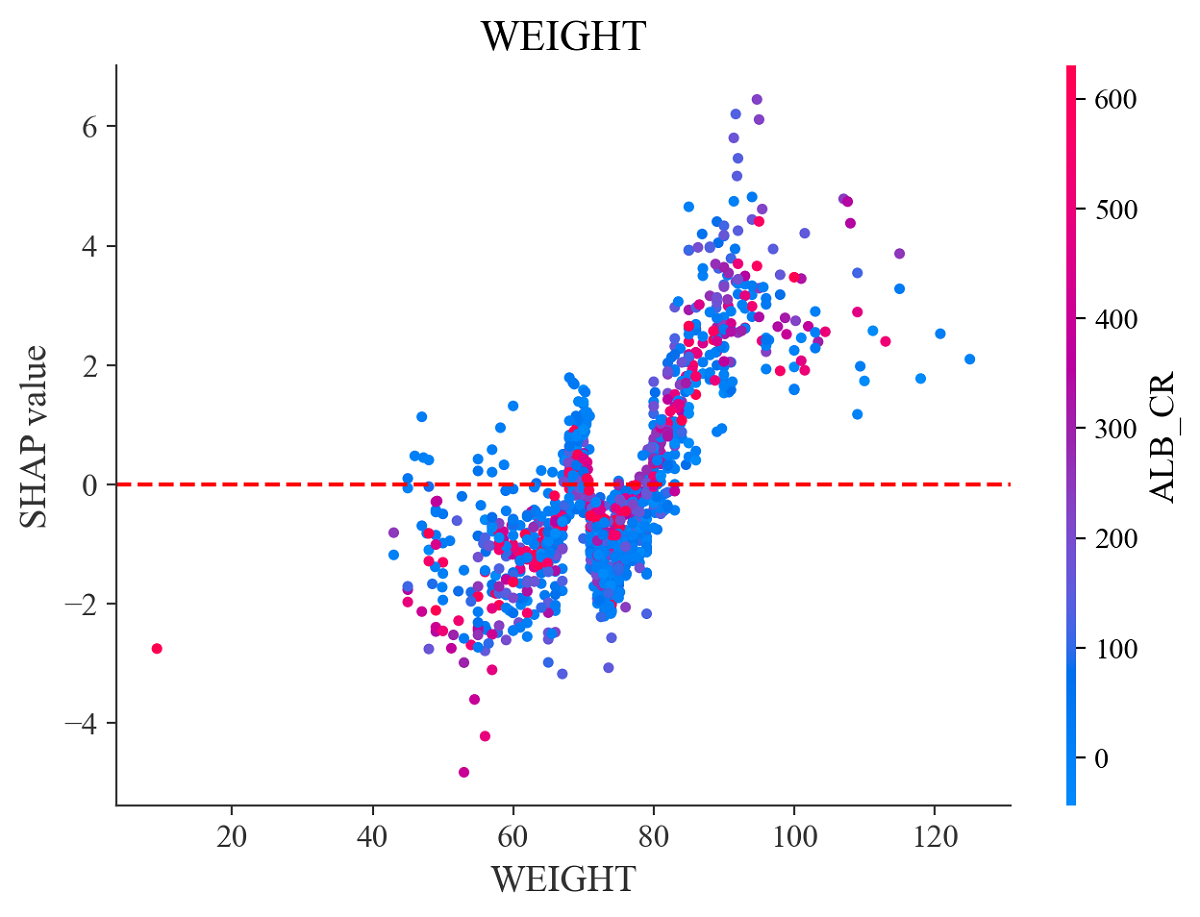

Supplement: Multimedia Appendix 4 [file medinform-v13-e64979-s004.png]

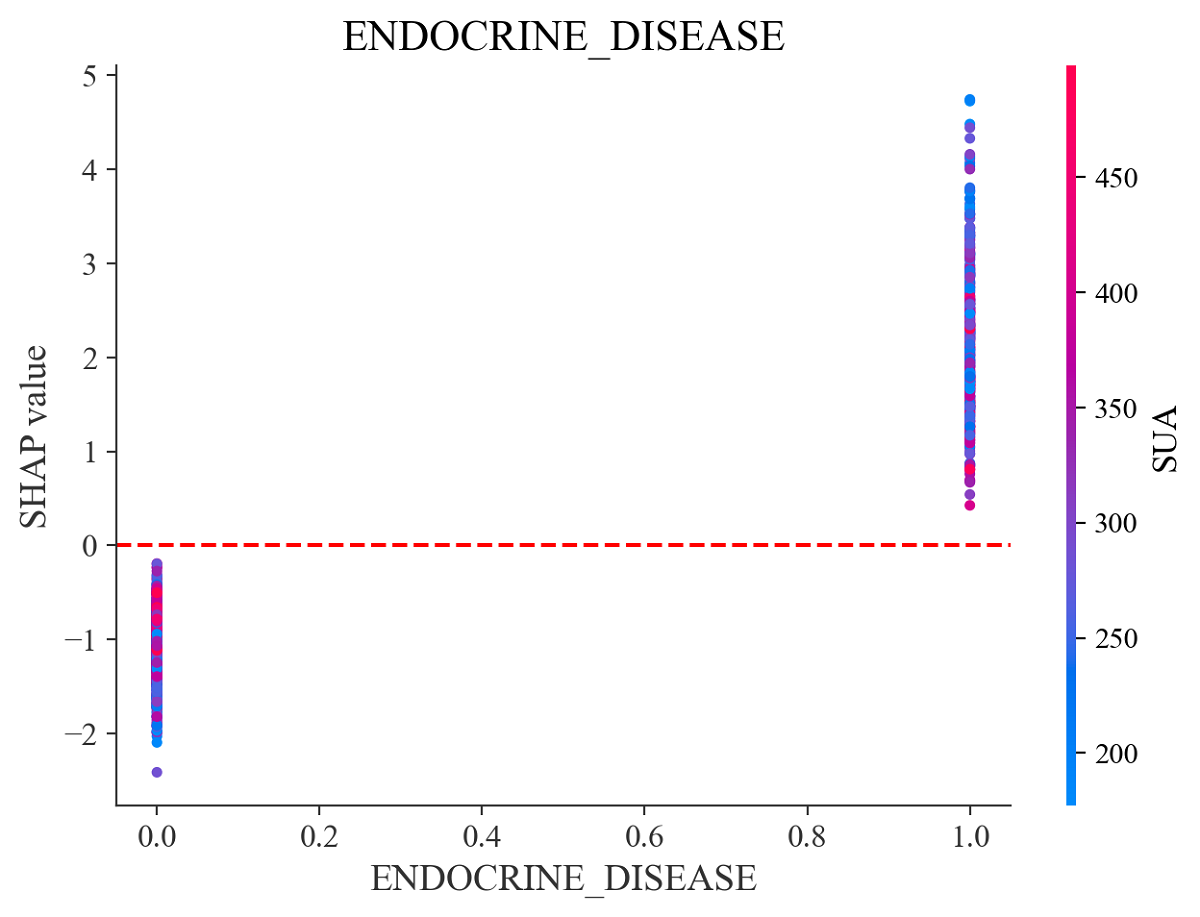

Supplement: Multimedia Appendix 5 [file medinform-v13-e64979-s005.png]

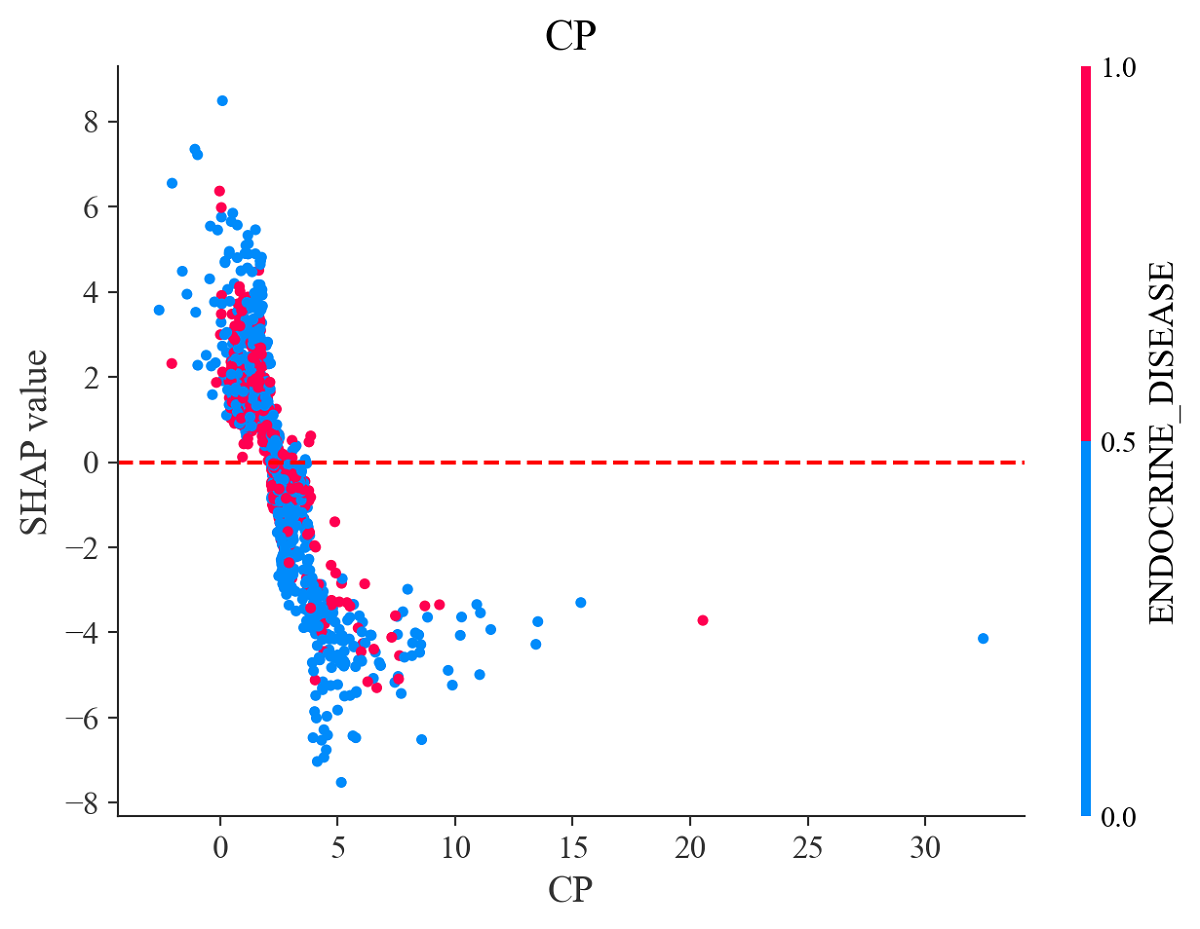

Supplement: Multimedia Appendix 6 [file medinform-v13-e64979-s006.png]

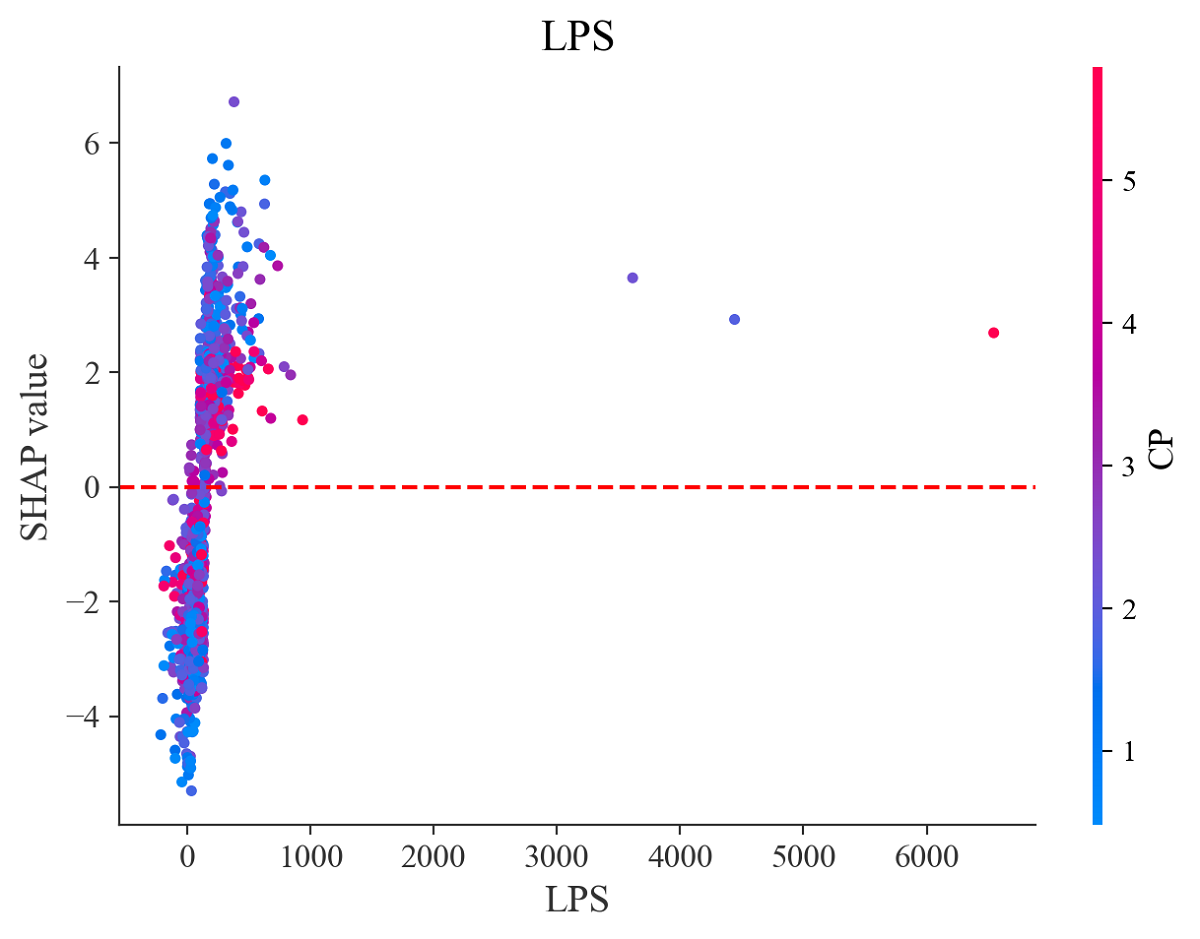

Supplement: Multimedia Appendix 7 [file medinform-v13-e64979-s007.png]

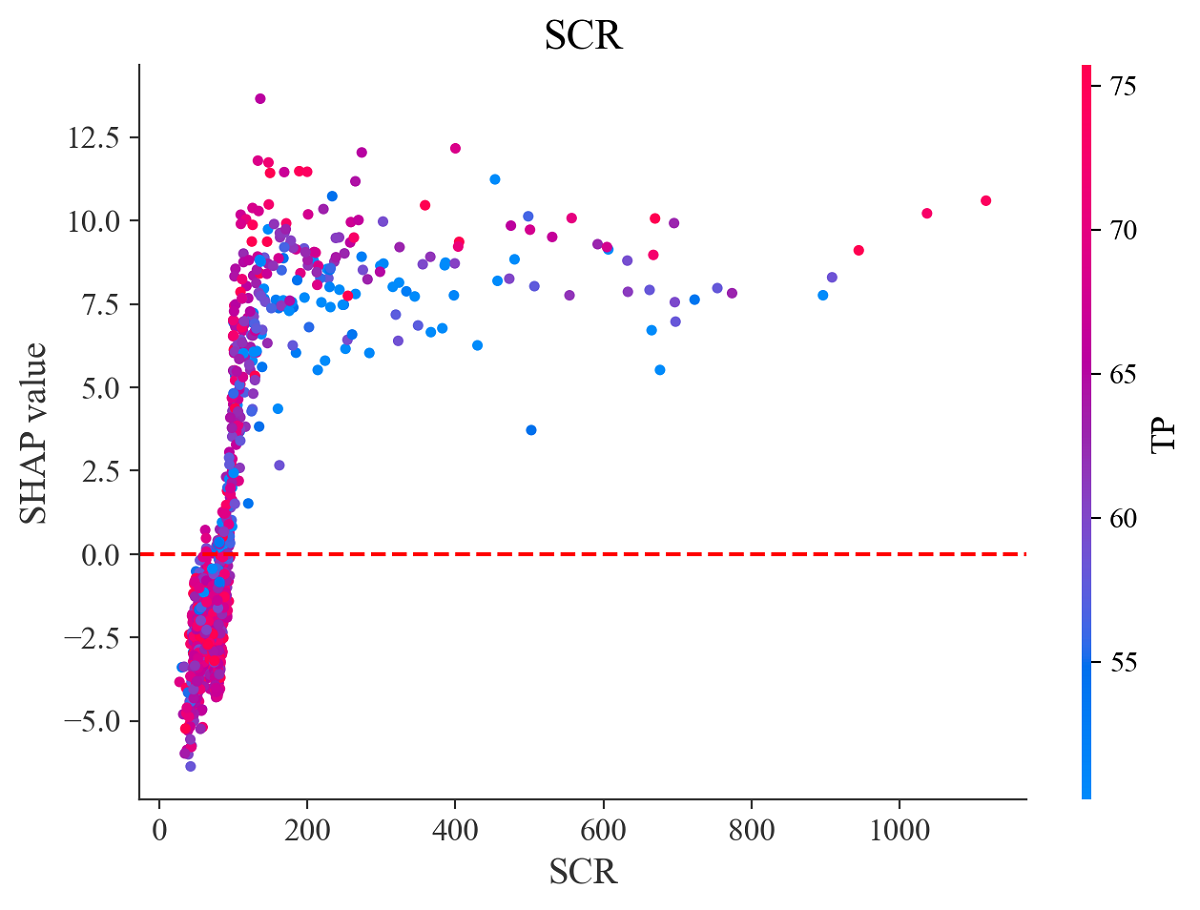

Supplement: Multimedia Appendix 8 [file medinform-v13-e64979-s008.png]

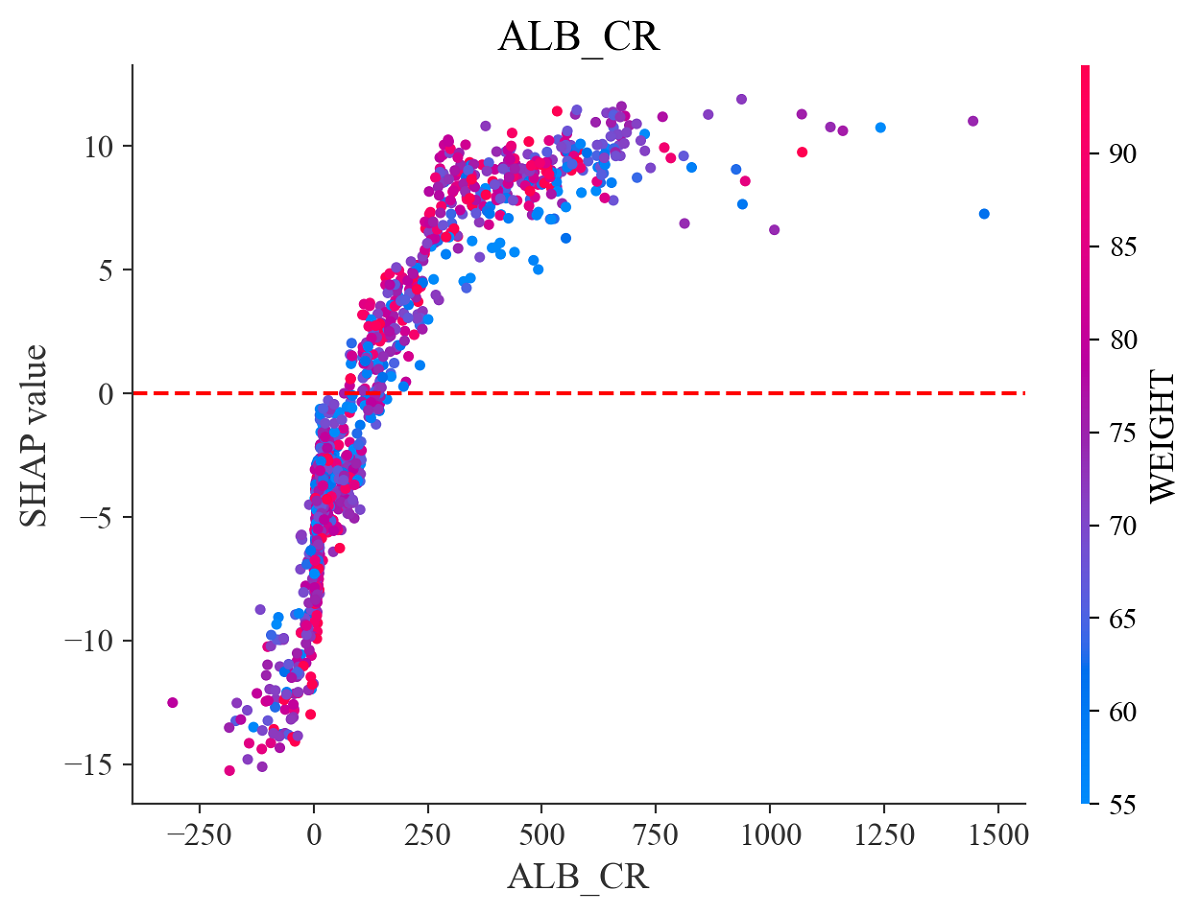

Supplement: Multimedia Appendix 9 [file medinform-v13-e64979-s009.png]
